# -1 Programmed ribosomal frameshifting in Class 2 umbravirus-like RNAs uses multiple long-distance interactions to shift between active and inactive structures and destabilize the frameshift stimulating element

**DOI:** 10.1093/nar/gkad744

**Published:** 2023-09-23

**Authors:** Anna A Mikkelsen, Feng Gao, Elizabeth Carino, Sayanta Bera, Anne E Simon

**Affiliations:** Department of Cell Biology and Molecular Genetics, University of Maryland, College Park, MD 20742, USA; Department of Cell Biology and Molecular Genetics, University of Maryland, College Park, MD 20742, USA; Department of Cell Biology and Molecular Genetics, University of Maryland, College Park, MD 20742, USA; Department of Cell Biology and Molecular Genetics, University of Maryland, College Park, MD 20742, USA; Department of Cell Biology and Molecular Genetics, University of Maryland, College Park, MD 20742, USA

## Abstract

Plus-strand RNA viruses frequently employ -1 programmed ribosomal frameshifting (-1 PRF) to maximize their coding capacity. Ribosomes can frameshift at a slippery sequence if progression is impeded by a frameshift stimulating element (FSE), which is generally a stable, complex, dynamic structure with multiple conformations that contribute to the efficiency of -1 PRF. As FSE are usually analyzed separate from the viral genome, little is known about *cis-*acting long-distance interactions. Using full-length genomic RNA of umbravirus-like (ula)RNA citrus yellow vein associated virus (CY1) and translation in wheat germ extracts, six tertiary interactions were found associated with the CY1 FSE that span nearly three-quarters of the 2.7 kb genomic RNA. All six tertiary interactions are conserved in other Class 2 ulaRNAs and two are conserved in all ulaRNAs. Two sets of interactions comprise local and distal pseudoknots that involve overlapping FSE nucleotides and thus are structurally incompatible, suggesting that Class 2 FSEs assume multiple conformations. Importantly, two long-distance interactions connect with sequences on opposite sides of the critical FSE central stem, which would unzip the stem and destabilize the FSE. These latter interactions could allow a frameshifting ribosome to translate through a structurally disrupted upstream FSE that no longer blocks ribosome progression.

## Graphical Abstract

**Graphical Abstract:**
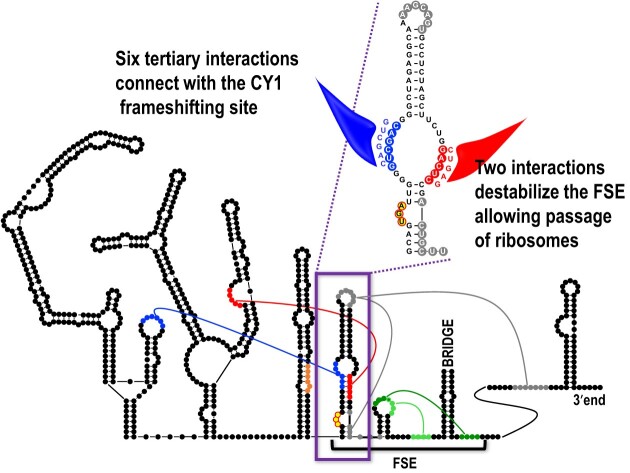

## INTRODUCTION

Eukaryotic RNA viruses employ different mechanisms to maximize the coding capacity of their genomes and produce the required stoichiometry of translated products. One such mechanism is programmed ribosomal frameshifting (PRF), which occurs when translating ribosomes encounter an impediment while associated with a slippery sequence and slip backward or forward into an alternate reading frame that bypasses the frame 0 termination codon (1–3). The most common type of PRF is a backward slip of 1 nt (-1 PRF), which transpires in many genera of plant and animal viruses. For monopartite RNA viruses, sequences that cause -1 PRF are frequently located near the termination codon of the 5′ proximal open reading frame (ORF), leading to translation of a replication-required protein such as the RNA-dependent RNA polymerase (RdRp). Since a precise concentration of RdRp is critical for virus fitness, even small changes in the efficiency of -1 PRF can have a significant negative effect on virus infectivity (4). The efficiency of viral frameshifting varies widely, and is usually very low (1–5%) in plant viruses (5,6) and up to 80% in animal viruses (7).

The minimal frameshifting element (MFE) is composed of a heptanucleotide slippery sequence, a spacer region and a structured frameshift stimulating element (FSE) (8). The slippery sequence generally consists of X XXY YYZ with X denoting identical nucleotides, Y is A or U, and Z is any nucleotide except G. -1 PRF occurs when two transfer RNAs (tRNAs) with modifications that enhance the flexibility of their anticodon loops (9) occupy the slippery sequence in the ribosome P- and A-sites. Following ribosome slippage, the tRNAs occupy the -1 frame XXX YYY codons in the P- and A-sites. Although more unusual, non-identical combinations of X can also be slippery, as long as interactions between the tRNAs and the template in the -1 frame are thermodynamically stable (10). The spacer sequence downstream of the slippery site can be 1 to 12 nt (usually 5 to 9 nt), a distance that allows the FSE to abut the ribosome at the messenger RNA (mRNA) channel, resulting in either ribosome stalling (11) or hindered A-site tRNA binding (12). Sequences and structures upstream of the MFE can also contribute to frameshifting efficiency and/or are phylogenetically conserved, including a nearby stem-loop structure in coronaviruses known as the attenuator hairpin, which is similarly present in plant umbraviruses (2,13–15). Although *cis-*acting elements are considered the primary determinants for efficiency (16), other factors can also influence frameshifting such as co-translational folding of the nascent polypeptide chain (17), cellular or viral-encoded transacting factors such as proteins (1,4,18,19) and possibly microRNAs (20), concentration of the A-site tRNA (21), as well as other facets of the cellular environment (8). These additional influences likely keep frameshifting rates from being static, thus allowing the ratio of terminated and extended products to vary to meet viral requirements during infection (18).

FSEs with diverse structural features have been reported for a number of different viruses (22). Structures are generally complex, containing at least one stem-loop along with one or more local pseudoknots, and have been shown or suggested to be dynamic and assume alternative conformations (23–25). Despite FSEs being studied for >30 years, how the FSE and other components of -1 PRF contribute to the efficiency of frameshifting is still not clear (22). Although an assumption for many years, frameshifting efficiency is not related to the thermodynamic stability of the entire FSE structure (26) although stabilizing base-pairs in the lowest stem enhances efficiency (26). Using an optical tweezer to study conformational changes of individual FSEs subjected to mechanical force, others have suggested that high efficiency -1 PRF correlates with increased plasticity of the structure, i.e. the ability of the FSE to assume multiple conformations (25,27,28).

One missing piece of the FSE puzzle is a lack of understanding of what constitutes a complete FSE structure, i.e. one that includes all *cis-* (and/or *trans*-) acting interactions and how these interactions contribute to active and inactive FSE conformations and frameshifting efficiency. An additional overlooked issue is how the frameshifted ribosome can resume translation through an FSE structure sufficiently stable to have caused the ribosome to pause. Problematically, FSE have been mainly studied following excision and integration into reporter constructs, which eliminates any contributions of distal sequences that might positively or negatively affect frameshifting efficiency (8). This issue was recently illustrated for the well-studied FSE of SARS-CoV-2; a three-stemmed pseudoknotted form that was proposed when short segments were analyzed (15,29) was absent when the FSE was studied within the full-length viral genome in infected cells (30). Examination of individual SARS-CoV-2 genomic RNAs suggested that the FSE assumes two equal molar conformations in living cells, neither of which contains a pseudoknot, with one conformation composed of sequences located 1.1 kb distal from the core element (30,31). The HIV-1 frameshift domain within packaged HIV-1 genomes is also larger, more complex and more dynamic than the originally characterized FSE (23). In plant luteoviruses and umbraviruses, long-distance interactions connect the FSE with sequences proximal to the 3′ end that are thousands of bases downstream, but how this long-distance interaction contributes to frameshifting is not known (5,32). These findings and others are leading to a growing consensus that FSEs need to be examined within their native context to fully understand how they function (4,22,30).

Umbravirus-like (ula)RNAs are a recent grouping of subviral RNAs with monopartite, plus-strand genomes of 2.7 to 4.5 kb. ulaRNAs code for at least two proteins; a 5′ proximal product (ORF1) that corresponds to a replication-required protein in related viruses, and the viral RdRp (33), which is generated by -1 PRF extension of the ORF1 protein. ulaRNAs share conserved RdRp sequences and 3′ terminal RNA features with umbraviruses, and both lack encoded capsid proteins. However, only ulaRNAs have been found in natural hosts in the absence of a helper virus (33–36).

**Figure 1.**
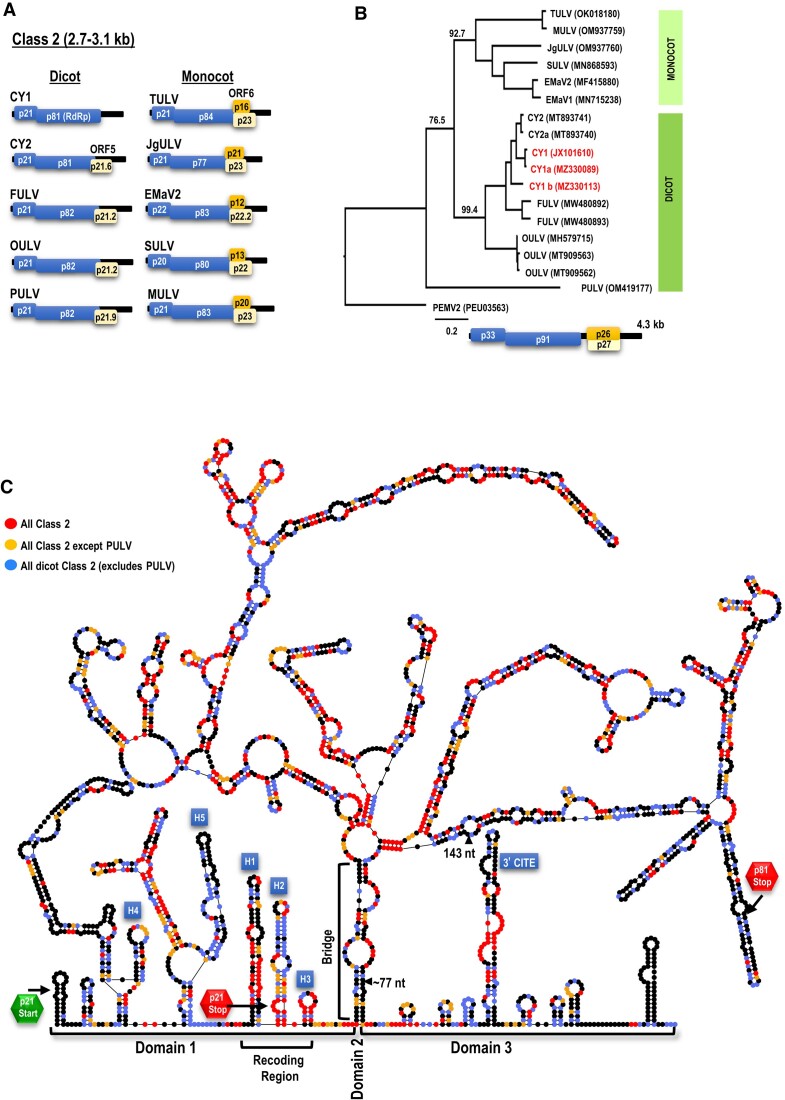
Class 2 ulaRNAs. A. Gene organization of Class 2 ulaRNAs. All ulaRNAs encode their own RdRp as a -1 PRF extension of the 5′ proximal ORF and all except CY1 have an additional ORF (ORF5). Monocot-infecting Class 2 ulaRNAs have an extra embedded ORF (ORF6) of different lengths. B. Maximum likelihood phylogenetic tree based on RdRp amino acid sequences. Branch numbers indicate bootstrap support in percentage out of 1000 replicates. The scale bar denotes amino-acid substitutions per site. Umbravirus PEMV2 was used as an outgroup and rooted to it. Dicot-infecting and monocot-infecting Class 2 ulaRNAs separate into different clades with the exception of parsley umbra-like virus (PULV), which occupies its own clade. CY1, CY1a and CY1b were isolated from greenhouse-grown citrus grafted with material from the same original infected Southern California limequat trees (34) and all lack ORF5. CY2 is closely related to CY1 and was isolated from hemp. C. Nucleotide sequence similarity among Class 2 ulaRNAs. The shown secondary structure of CY1 divides into three domains as indicated. The ‘Bridge’ that separates Domains 1 and 3 forms from the lower stem of Domain 2. The 3′ cap-independent translation enhancer (CITE) is labeled as are the hairpins (H1 to H5) that are referred to in this report. Each colored ‘dot’ is a nucleotide and color code reflects conservation of the nucleotide as designated in the figure. Triangles denote large deletions missing in all CY1s. CY1, citrus yellow vein associated virus (also known as CYVaV); CY2, citrus-yellow vein associated virus-isolate delta; FULV, fig umbra-like virus (51); OULV, opuntia umbra-like virus (35); EMaV1 and 2, Ethiopian maize-associated virus; Teosinte umbra-like virus (TULV) (52), Maize umbra-like virus (MULV) (53), Johnson grass umbra-like virus (JgULV) (53), Sugarcane umbra-like virus (SULV) (54) and parsley umbra-like virus (PULV) (55). Accession numbers are shown.

Phylogenetic analysis of ulaRNAs suggest that they separate into three classes (Supplementary Figure S1A): Class 1 do not encode any additional proteins and have an extensive 3′ UTR (untranslated region); Class 3 have one or two additional unique ORFs that partially overlap the RdRp ORF (Supplementary Figure S1B) and Class 2 members that infect dicot hosts contain one additional ORF (ORF5) that also partially overlaps the RdRp ORF, and members infecting monocots have an additional ORF (ORF6) of different sizes that is embedded in ORF5 (Figure 1A).

An unusual member of the dicot-infecting Class 2 ulaRNAs is citrus yellow vein-associated virus (CYVaV or CY1; 2.7 kb). Unlike other Class 2 ulaRNAs, CY1 is missing two large fragments that together with additional alterations eliminate ORF5 (33). Using selective 2′ hydroxyl acylation analyzed by primer extension (SHAPE) RNA structure probing along with phylogenetic structural comparisons among Class 2 ulaRNAs, the complete 2.7 kb secondary structure of CY1 was previously solved (Figure 1C) (33). The structure organizes itself into three domains: Domain 1 encompasses the 5′ end through position 668, including the entire ORF1; Domain 3 extends from position 2399 to the 3′ end and includes a portion of the 3′ UTR; Domain 2 extends from residues paired at the base of the domain that are located 1.7 kb apart, forming the lower Domain 2 stem known as the ‘Bridge’ that juxtaposes Domains 1 and 3.

The frameshifting efficiency of full-length CY1 assayed in wheat germ extracts (WGE) is unusually high for a plant virus at 29%, nearly 6-fold higher than that of umbravirus pea enation mosaic virus 2 (PEMV2) (33). The CY1 MFE was originally proposed to be fully contained upstream of the Bridge in Domain 1 (33), however, deletion analyses that led to the identification of the CY1 3′ cap-independent translation enhancer (3′CITE) suggested that elements involved in -1 PRF also exist in several locations on the 3′ side of the Bridge in Domain 3 (37). To further explore this unusually efficient frameshifting element, interrogation features within the RNAcanvas structure drawing web app (https://rna2drawer.app/) (38) were used to identify short- and long-distance tertiary interactions that are conserved among Class 2 members. We report here the discovery of six tertiary interactions between Class 2 FSEs and sequences spanning nearly three-quarters of the ulaRNA genomes, some of which are also conserved in Class 1 and Class 3 ulaRNAs. Two sets of interactions comprise local and distal pseudoknots that are structurally incompatible and are proposed to help the FSE shift between (and stabilize) inactive and active structures. Importantly, two long-distance interactions involve sequences on opposite sides of the critical middle stem of the main FSE hairpin, which when present would unzip this critical stem. We propose that destabilizing the FSE accompanies frameshifting, contributing to ribosome progression in the -1 frame through the region that was responsible for the initial ribosome stalling.

## MATERIALS AND METHODS

### Construction of CYVaV mutants

Plasmid pET17b-CYVaV, which contains the entire length of WT CYVaV genomic RNA (gRNA) complementary DNA (Accession number JX101610) downstream of a T7 promoter (33), was used as a template for polymerase chain reaction (PCR)-based site-directed mutagenesis. The desired mutations were introduced using custom-designed oligonucleotide primers (Integrated DNA Technologies) using Q5 high-fidelity DNA polymerase (New England Biolabs). The resulting PCR products were subjected to DpnI digestion followed by T4 DNA ligase before transformation into DH5α *Escherichia coli* cells. The presence of the desired mutations was confirmed through Sanger sequencing (Eurofins Genomics).

### *In vitro* transcription and translation

Uncapped gRNA transcripts were generated by *in vitro* transcription using bacteriophage T7 RNA polymerase and pET17-b CY1 plasmids linearized with HindIII. Synthesized RNA was quantified using a DeNovix DS-II FX spectrophotometer and the quality of RNA was verified on a 1% TBE native agarose gel. Uncapped *in vitro* transcribed RNA (0.5 pmol) was translated in 10 μl of WGE with ^35^S methionine as per the manufacturer's (Promega) recommendations. Briefly, RNA was denatured at 65°C for 5 minutes, followed by snap cooling in ice. The translation mixture was incubated at 25°C for 45 minutes and then resolved in a 10% SDS–PAGE gel. After drying, the gel was exposed to a phosphorus screen and scanned by an Amersham Typhoon fluorescent image 166 analyzer. %PRF was determined by quantifying radiolabeled bands using ImageJ software (39) using the following equation: [p81 / (p81 + p21)] • 100 and taking into account the number of radiolabeled methionines in p21 and p81 (1:7). All experiments were independently repeated at least three times and final %PRF was the average of the repeat values. Statistical analysis and graphing were performed using GraphPad Prism v.9.

### Protoplast transfection and viral RNA detection

Arabidopsis callus was generated from sterilized seeds in 1.0% Murashige and Skoog supplemented with 2 mg/ml kinetin and 2 mg/ml 2,4-D and incubated in a growth chamber at 20°C as previously described (40,41). Protoplasts were prepared from white friable callus (42) and 5 × 10^6^ protoplasts were transfected with 10 μg of CY1 gRNA transcripts followed by incubation at 22°C for 24 h in the dark. Total RNA was extracted using RNA extraction buffer (50 mM Tris-HCl [pH 7.5], 5 mM EDTA [pH 8.0], 100 mM NaCl and 1% SDS), followed by phenol-chloroform extraction and 3 M sodium acetate ethanol precipitation. Five micrograms of total RNA were subjected to 1.5% agarose electrophoresis, transferred to nitrocellulose and probed with α^32^P labeled oligonucleotides complementary positions 248–262, 390–429, 1029–1068, 1974–2013, 2202–2239, 2464–2502 and 2655–2692 nt. The blot was exposed to a phosphorous screen and scanned in an Amersham Typhoon fluorescent image 166 analyzer. The band's intensity was quantified using ImageJ software (39).

### RNA structure drawing

All RNA structures were drawn using RNAcanvas (https://rna2drawer.app/) (38).

## RESULTS AND DISCUSSION

### CY1 requires a UGA termination codon for -1 PRF

**Figure 2.**
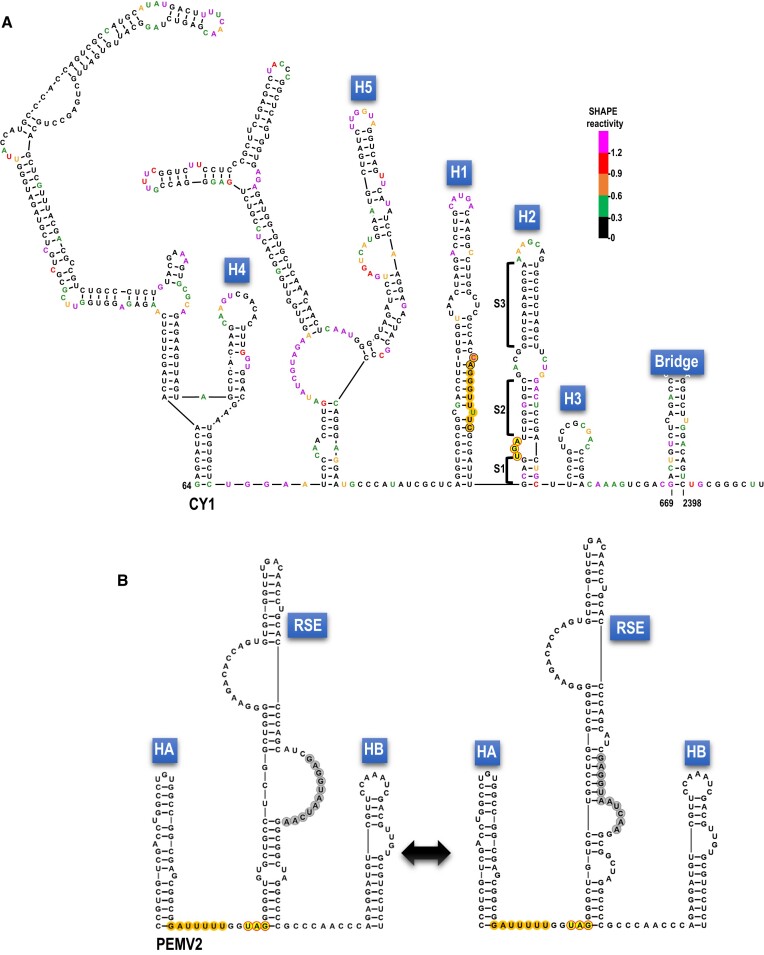
Conserved structure in the vicinity of the recoding region of Class 2 ulaRNAs. A. Structure shown is from CY1. Additional structures for some Class 2 ulaRNAs are shown in Supplementary Figure S2. Nucleotide colors denote SHAPE reactivity for full-length CY1 transcripts synthesized *in vitro* (33). Note that the SHAPE data does not support the lower stems (S2 and S1) of hairpin H2. Hairpin labels and the Bridge are described in legend to Figure 1C. Slippery site with three additional conserved nucleotides (circled) are in orange. Stop codon for the first ORF is in yellow with a red border. Numbers reflect nucleotide positions in CY1. B. Recoding region of umbravirus PEMV2. The structure on the left is from (32). The structure on the right is an alternative structure conserved in some umbraviruses. Nucleotides shaded in gray participate in a long-distance interaction with residues in the apical loop of the 3′ terminal hairpin.

Figure 1C depicts nucleotide sequence conservation among Class 2 ulaRNAs using a full-length CY1 secondary structure as a template. The recoding region, located just 5′ of the Domain 2 Bridge, is well conserved in this class of ulaRNAs with the exception of the upper and lower portions of hairpin H1 (Figure 2A). Within the 3′ UTR, two regions are highly conserved: the middle portion of the 3′ CITE, and the 5′ end of Domain 3, which is just across the Bridge from Domain 1. Previous deletions in CY1 that included the 5′ end of Domain 3 reduced frameshifting in WGE to background levels (37), suggesting that the recoding region extends across the Bridge into Domain 3.

Figure 2A depicts one conformation of the recoding region that is conserved in all Class 2 ulaRNAs (Supplementary Figure S2) together with previously generated (33) *in vitro* SHAPE structure probing data. As with umbraviruses (Figure 2B), there are three hairpins (H1, H2 and H3) in the vicinity of the p21 (ORF1) UGA stop codon. The slippery sequence (G GGU UUU) is contained within H1, thus differing from the separate locations of the slippery sequence and 5′ HA hairpin in umbraviruses (Figure 2B). The CY1 slippery sequence is conserved in all Class 2 ulaRNAs along with upstream CA residues and a downstream C residue (Supplementary Figure S1C). SHAPE data are consistent with the conformation of H1 shown in Figure 2A, but not with the lower (S1) and middle (S2) stems of downstream hairpin H2. This suggests that a significant portion of *in vitro* synthesized CY1 transcripts only contain the H2 upper stem (S1). The small H3 stem-loop is highly conserved in Class 2 ulaRNAs with 11 of 19 identical residues and four additional residues conserved in all dicot-infecting Class 2 ulaRNAs (Figure 1C).

**Figure 3.**
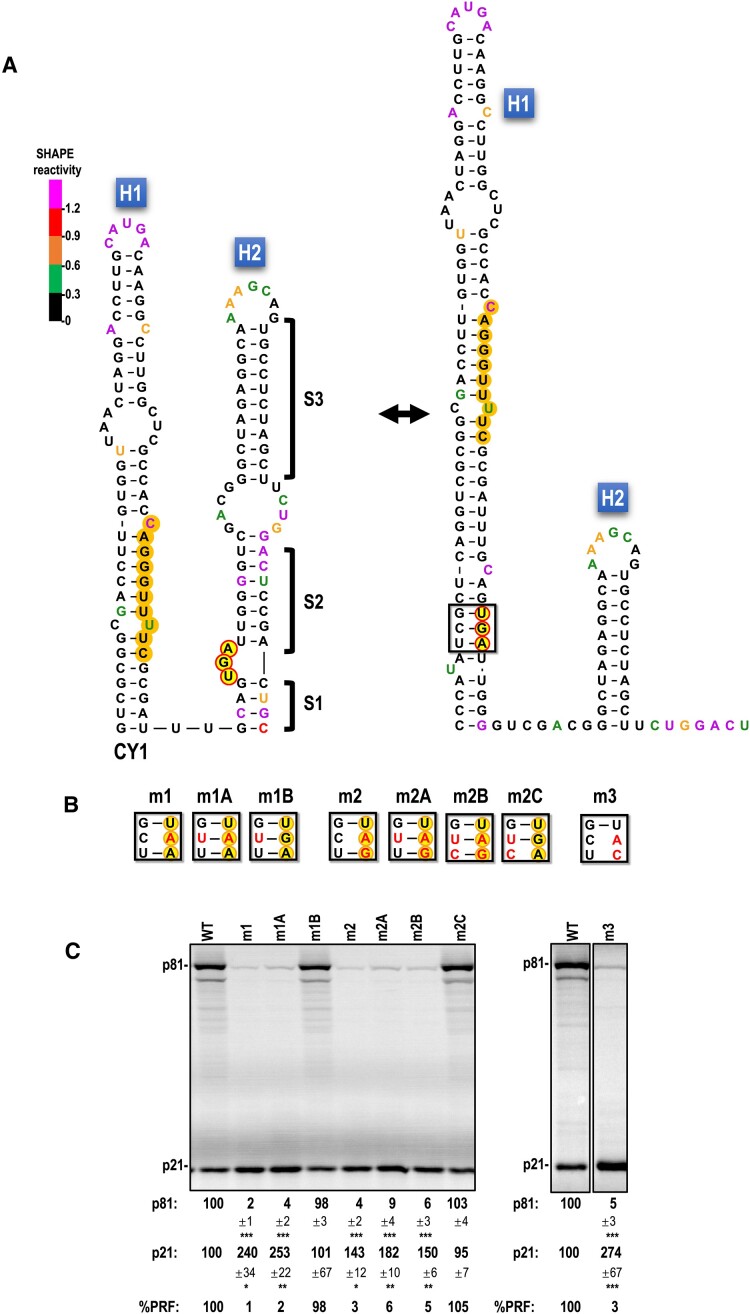
CY1 frameshifting requires a UGA termination codon. A. Alternative structure for CY1 H1/H2. The two structures shown are conserved in all Class 2 ulaRNAs (Supplementary Figure S3). SHAPE color coding of nucleotides and shading are as described in the caption to Figure 2. Note that SHAPE data more closely support the H1/H2 structure on the right. B. Full-length CY1 transcripts were altered to convert the UGA termination codon to either UAA or UAG, with and without additional alterations to maintain base-pairing of the termination codon in the alternative conformation of H1. Base changes are in red. C. *In vitro* translation of full-length WT CY1 (WT) or mutant transcripts in WGE. Data are averages from at least three independent experiments and are normalized to levels of WT translation. Standard deviations are shown. One-way ANOVA was performed using GraphPrism software. *, ** and *** are *P* values of 0.01, <0.01 and <0.0001, respectively.

Seven out of 10 Class 2 ulaRNAs have a UGA termination codon identically positioned downstream of the slippery site, which occupies a bulge loop in the conformation of H2 shown in Figure 2A. Three Class 2 ulaRNAs have a UAG termination codon two codons upstream of an identically positioned UGA triplet (Supplementary Figures S1C and S2). Class 1 and 3 ulaRNAs have either a UGA or UAA stop codon at the same position relative to the slippery sequence as CY1, with the exception of Class 1 papaya meleira virus 2 (PMeV2), whose UAG stop codon is immediately downstream of the slippery site. In addition to the H1/H2 conformation shown in Figure 2A, an additional conformation is phylogenetically conserved in Class 2 ulaRNAs that place the UGA termination codon within H1 by pairing sequence upstream of H1 with sequences on the 5′ side of S1 (and the UGA), which extends the H1 lower stem (Figure 3A). In support of this additional conformation are several covariant base-pairs and base differences in other Class 2 ulaRNAs that maintain similar H1/H2 conformations (Supplementary Figure S3).

To determine whether the general conservation of a UGA stop codon in Class 2 ulaRNAs reflects a frameshifting requirement and/or a need for base-pairing in the alternative H1/H2 conformation, mutations were generated in CY1 that changed the stop codon to UAA or UAG while reducing or maintaining base-pairing (Figure 3B). Changing the wild-type (WT) UGA stop codon to UAA in the absence or presence of an additional alteration that maintains base-pairing (m1 and m1A, respectively) reduced frameshifting to background levels (25- to 50-fold), which enhanced synthesis of the ORF1 p21 protein (Figure 3C). CY1 containing the WT UGA and just the additional m1A alteration (C to U) frameshifted at WT levels (m1B), suggesting that this alteration does not by itself contribute to the reduction in m1A frameshifting. A similar set of alterations were generated to introduce a UAG stop codon in place of the WT UGA. Transcripts containing only the UGA to UAG alteration (m2) frameshifted at background levels as did constructs that included one (C to U) or two (CU to UC) additional changes to maintain base-pairing of the UAG in the alternative H1/H2 conformation (m2A and m2B). WT CY1 containing the m2B CU to UC alterations, which reduces UGA base-pairing in the alterative conformation, did not significantly affect frameshifting (m2C), suggesting that the conserved UGA base-pairing in the alternative H1/H2 conformation is not required in the active form of the recoding region. Alteration of the UGA to a non-stop codon (UAC) also reduced frameshifting to background levels (m3). In this construct, translation of p21 terminates 6 codons downstream at an in-frame UAG. These results strongly indicate that a UGA stop codon is necessary for efficient CY1 -1 PRF in WGE.

### H2, H3 and the Bridge are important for efficient CY1 -1 PRF

**Figure 4.**
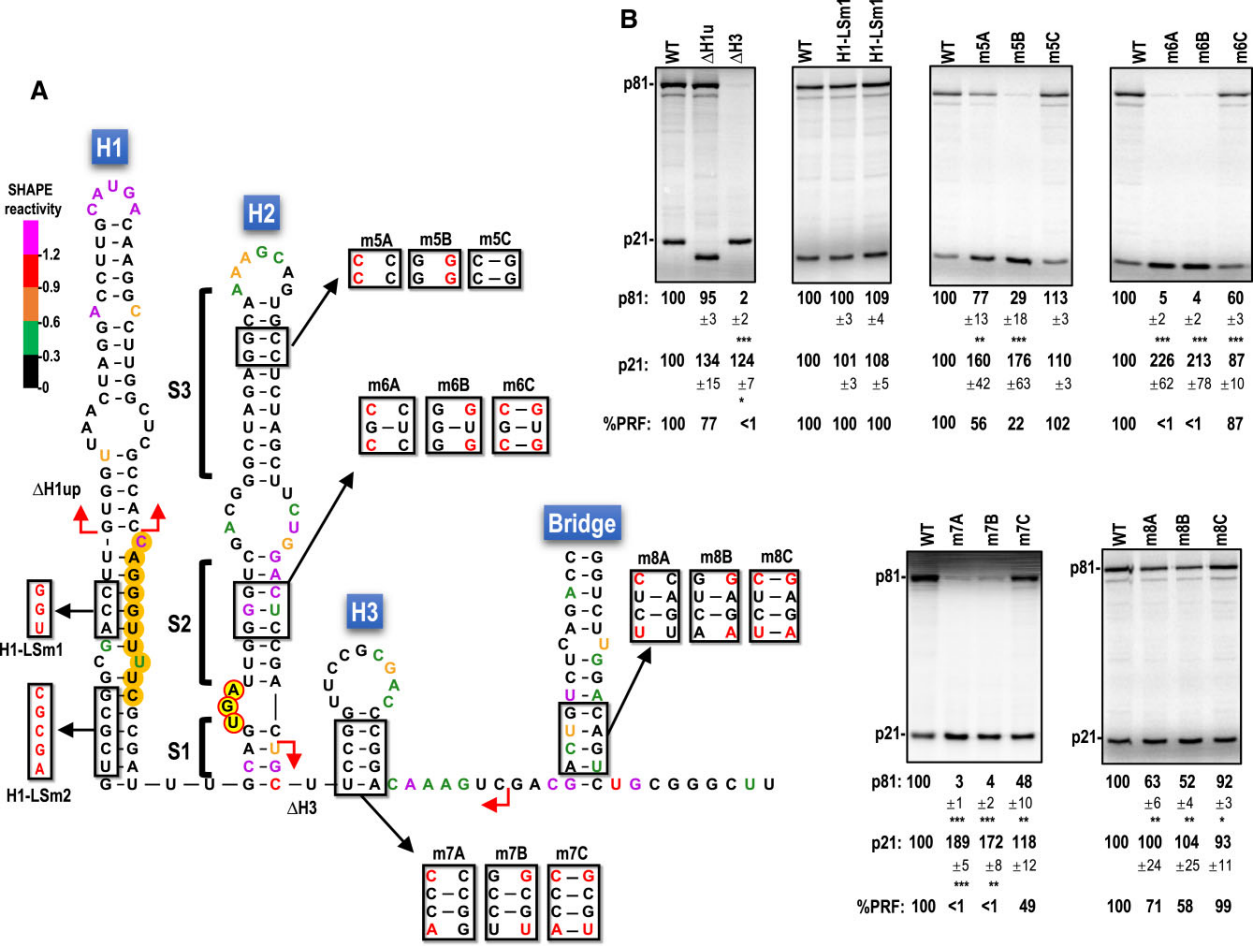
H2 and H3 in the CY1 FSE are critical for efficient frameshifting. A. CY1 structure in the vicinity of the recoding site with SHAPE structure probing data. Location of alterations in full-length CY1 transcripts are shown. Altered residues are in red. Other color shadings are as described in the caption to Figure 2A. Red arrows denote deletion end points. B. *In vitro* translation of full-length WT CY1 (WT) or mutant transcripts in WGE. Data are averages from at least three independent experiments and are normalized to levels of WT translation. Standard deviations are shown. One-way ANOVA was performed using GraphPrism software. *, ** and *** are *P* values of 0.01, <0.01 and <0.0001, respectively. PRF levels are normalized to WT CY1.

To examine other components of the Domain 1 recoding region, deletions and/or mutations were generated in H1, H2, H3 and the Domain 2 Bridge (Figure 4A). Deletion of the poorly conserved upper half of H1 (ΔH1up) reduced frameshifting by 23%, while extensive alterations in the lower H1 stem (H1-LSm1, H1-LSm2) had no effect on frameshifting (Figure 4B). By contrast, deletion of H3 along with four upstream residues and five downstream residues (30 nt) that disrupt the lowest (S1) stem of H2 (ΔH3) reduced -1 PRF to background levels. Mutations that only disrupt the H3 stem also reduced frameshifting to background levels (m7A and m7B), which was partially restored (49% of WT) when both sets of mutations, designed to be compensatory, were present (m7C). This result is in contrast with deletion of the similarly situated hairpin in PEMV2, which could be deleted without affecting frameshifting in WGE (13) Mutations in the upper (S3) stem of H2 (m5A and m5B) reduced frameshifting (by 44 and 78%, respectively), which was restored to WT levels when both sets of mutations reestablished the S3 stem (m5C). The H2 S2 stem, which was not structurally supported by SHAPE data, was nevertheless critical for frameshifting; pairs of mutations disrupting S2 reduced frameshifting to background levels (m6A, m6B), whereas together, the compensatory changes that should reform S2 increased frameshifting to 87% of WT. This latter result suggests that S2 is present and critical in the active FSE conformation and that SHAPE data reflect that most *in vitro* synthesized transcripts are adopting an alternative, inactive structure.

The lower stem of the Bridge was also not well supported by SHAPE data. Mutations on either side of the Bridge (m8A, m8B) reduced frameshifting by 29 and 42%, respectively. Inclusion of both sets of alterations (m8C), which should reform the stem, enhanced frameshifting to WT levels. This result supports the presence of the long-distance pairing that forms the Bridge in the secondary structure of CY1 and its importance for efficient frameshifting.

### The apical loop of H2 participates in local and distal pairings that are structurally incompatible

**Figure 5.**
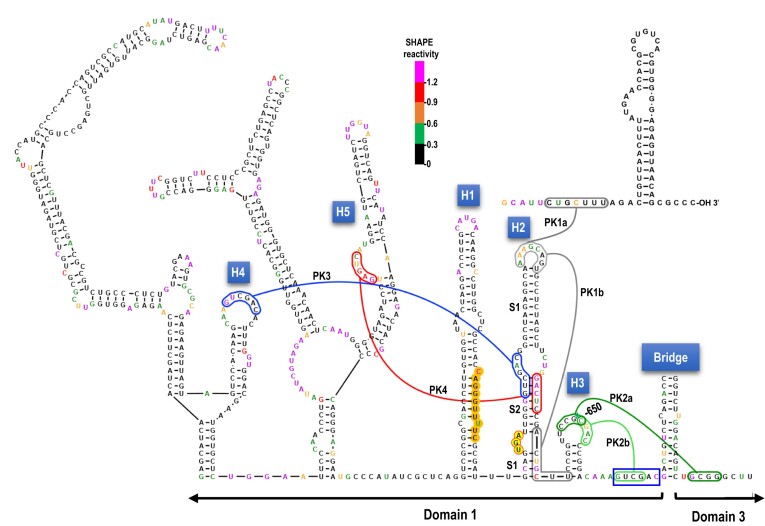
Six proposed tertiary interactions with the CY1 FSE. CY1 structure is presented with SHAPE structure probing data. Other color shadings are as described in the caption to Figure 2A. PK1 (gray) pairs bases in the apical loop of H2 with either a sequence proximal to the 3′ end (PK1a) or local H2 internal sequences (PK1b). PK2 (green) pairs bases in the apical loop of H3 with bases in Domain 3 just past the Bridge (PK2a, dark green). C650 (circled) along with three downstream bases also pair with bases just upstream of the Bridge in Domain 1 (PK2b, light green). PK3 (blue) pairs bases in the apical loop of H4 with sequence on the 5′ side of H2 and has a second possible pairing partner that overlaps PK2b sequences (blue box). PK4 pairs bases in the hairpin H5 interior bulge with a sequence on the 3′ side of H2. All these tertiary interactions are conserved in all Class 2 ulaRNAs (Supplementary Figure S4).

Efficient ribosome recoding in umbraviruses and other members of the *Tombusviridae*, whether -1 PRF or ribosome stop codon readthrough, requires a long-distance interaction between the apical loop or interior bulge loop of the recoding stimulating e with sequences in the: (i) apical loop of the 3′ terminal hairpin; (ii) apical loop of the 3′ penultimate hairpin or (iii) spacer sequence linking the two hairpins (5,6,32,43–46). Although ulaRNAs are not yet designated as members of the *Tombusviridae*, their close relationship with umbraviruses suggested that a similar long-distance tertiary interaction likely exists for CY1. Using the Pairing Function in RNAcanvas, a long-distance interaction similar to the one present in members of the *Tombusviridae* was found between seven residues in the CY1 H2 apical loop and the spacer sequence linking the two 3′ terminal hairpins (Figure 5, in gray, labeled PK1a). Surprisingly, the FSE apical loop sequence, minus one residue at the 5′ end and with one added residue at the 3′ end has a second, local pairing partner on the 3′ side of the lower portion of the H2 structure that includes S1 and extends through the first paired base in H3 (Figure 5, in gray, labeled PK1b). Whereas PK1a is compatible with the structure shown in Figure 5, PK1b would require an alternative conformation that lacks (at least) S1.

**Figure 6.**
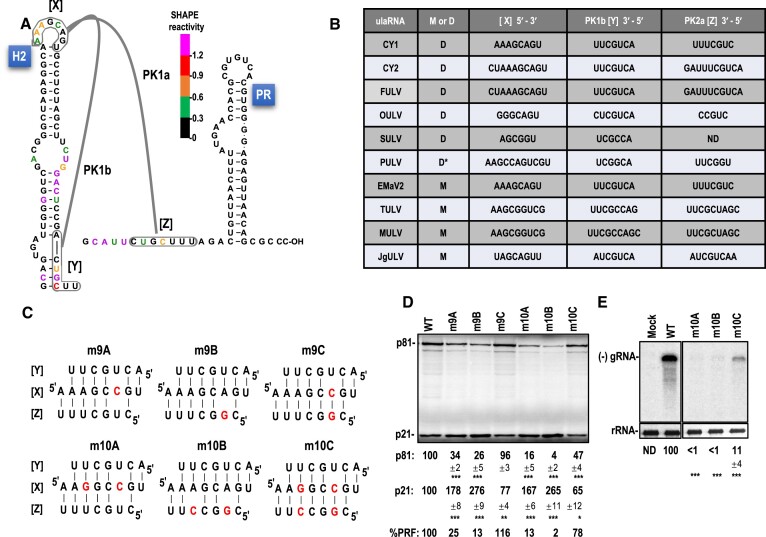
PK1a is important for efficient frameshifting. A. Proposed PK1a and PK1b tertiary interactions. For simplicity, H2 apical loop sequences, PK1b pairing sequences and PK1a pairing sequences are labeled [X], [Y] and [Z], respectively. Bases are colored by SHAPE structure probing. B. Table showing all Class 2 ulaRNA PK1 [X], [Y] and [Z] sequences. M or D denote whether the ulaRNA is monocot-infecting or dicot-infecting. Asterisk denotes that PULV occupies a different clade from the other dicot-infecting Class 2 members. The [Z] sequence is undetermined for SULV as the GenBank sequence terminates within the 3′ CITE. C. Mutations (in red) in [X] and [Z] sequences that were incorporated into full-length transcripts of CY1 and their effect on PK1a and PK1b. D. *In vitro* translation of full-length WT CY1 (WT) or mutant transcripts in WGE. Data are averages of at least three independent experiments and are normalized to levels of WT translation. Standard deviations are shown. One-way ANOVA was performed using GraphPrism software. *, ** and *** are *P* values of 0.01, <0.01 and <0.0001, respectively. E. WT and mutant CY1 minus-strand accumulation *in vivo*. *A. thaliana* protoplasts were transfected with WT CY1 or CY1 containing [X], [Z] and [X]+[Z] mutations and total RNA examined by RNA gel blots 24 h later using oligonucleotide probes that detect only the complementary minus-strands. Ribosomal (r)RNA was used as a loading control. Data are from three independent experiments.

The three components of this putative set of tertiary interactions (H2 apical loop sequence, H2 lower stem pairing partner in PK1b and Domain 3 pairing partner in PK1a) were designated as [X], [Y] and [Z], respectively (Figure 6A). All Class 2 ulaRNAs contain between 6 and 10 Watson–Crick base-pairs capable of forming PK1a and PK1b (Figure 6B; Supplementary Figure S4), strongly supporting the validity of both proximal and distal interactions. To further analyze these putative tertiary interactions, single and compensatory mutations were generated in CY1 [X] and [Z] sequences (Figure 6C) and their effects on -1 PRF were determined in WGE (Figure 6D). A single base change in [X] (m9A), which would disrupt both PK1a and PK1b, reduced frameshifting by 4-fold (Figure 6D). A single alteration in [Z] (m9B), which disrupts PK1a while maintaining PK1b, reduced frameshifting by 8-fold. Combining the two mutations, which should re-establish PK1a but not PK1b (m9C), restored frameshifting to at least WT levels. These results support the validity of PK1a and its importance in the active conformation of the FSE.

Since the single mutations still permit four (PK1a) or five (PK1b) paired bases, the experiment was repeated with the addition of a second mutation that should further weaken the putative interactions. m10A with two mutations in [X] that should affect formation of both PK1a and PK1b (Figure 6C), decreased frameshifting by 8-fold. The corresponding mutations in [Z], which should not affect PK1b but would more fully disrupt PK1a, caused a reduction in frameshifting to background levels. The four mutations together, which were compensatory, restored frameshifting to 78% of WT. These results strongly suggest that PK1a, but not PK1b, exists in an active FSE conformation. Because PK1b is positionally conserved in all Class 2 ulaRNAs, we propose that PK1b exists in an alternative, inactive conformation that would preclude formation of PK1a. Interestingly, PK1b is also conserved in all Class 1 and Class 3 ulaRNAs, unlike PK1a, which is only present in one of the Class 3 ulaRNAs (grapevine umbra-like virus; GULV) (Supplementary Figures S5 and S6). These results indicate that PK1a exists to maintain the FSE in an active conformation, and that unknown additional factors are likely needed to similarly prevent PK1b formation in Class 1 and most Class 3 ulaRNAs.

Mutations in the H2 apical loop disrupt PK1a without altering the RdRp amino acid sequence, allowing m10A, m10B and m10C to be assessed for effects on CY1 accumulation *in vivo*. Transcripts containing these mutations were transfected into *Arabidopsis thaliana* protoplasts and levels of CY1 minus strands were assessed 24 h later using RNA gel blots (Figure 6E). Mutations that disrupt PK1a (m10A and m10B) reduced accumulation to below the level of detection, while the compensatory m10C alterations enhanced accumulation to 11% of WT. This result supports the importance of PK1a while also suggesting that PK1b or other parameters associated with the WT pseudoknots is required for efficient accumulation. Altogether, these results, combined with PK1a and PK1b being conserved in all Class 2 ulaRNAs, support the importance of PK1a in the active RSE structure and also suggest that PK1b, which is incompatible with PK1a and present in all ulaRNAs, exists in an alternative, inactive conformation of CY1 that is necessary for efficient virus accumulation.

### The apical loop of CY1 H3 has local and distal pairing partners

**Figure 7.**
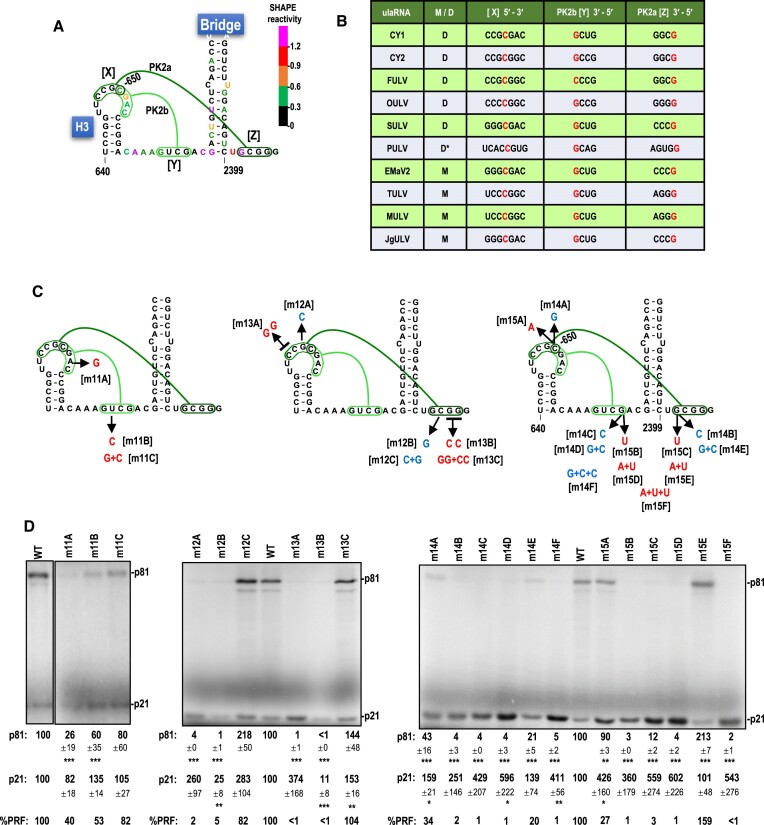
PK2a is more critical for frameshifting than PK2b. A. Proposed PK2a and PK2b tertiary interactions. Bases are colored by SHAPE structure probing. For simplicity, H3 apical loop sequences, PK2b pairing sequences and PK2a pairing sequences are labeled [X], [Y] and [Z]. B. Table showing all Class 2 ulaRNA PK2 [X], [Y] and [Z] sequences. With the possible exception of PULV, PK2a and PK2b interactions contain four base-pairs with one shared nucleotide (C650), and are found in identical locations in all Class 2 ulaRNAs. The nucleotide in red is C650 in the [X] sequence and pairing partners in the other two sequences. C. Mutations (in red and blue) that disrupt PK2a, PK2b or both. D. *In vitro* translation of full-length WT CY1 (WT) or mutant transcripts in WGE. Data are averages of at least three independent experiments and are normalized to levels of WT translation. Standard deviations are shown. One-way ANOVA was performed using GraphPrism software. *, ** and *** are *P* values of 0.01, <0.01 and <0.0001, respectively. PRF values are a percentage of the WT level.

A second set of tertiary interactions is proposed for the apical loop of hairpin H3 (Figure 5, in green). This set of interactions is positionally conserved for all Class 2 ulaRNAs, with many containing one or more co-variant base-pairings (Figure 7B; Supplementary Figure S4). One of these tertiary interactions is between the four residues just downstream of two conserved uridylates in the H3 loop and four residues located 1 nt downstream of the Bridge within Domain 3 (PK2a, dark green, Figure 7A). The only exception is PULV, an outlier among Class 2 ulaRNAs (Supplementary Figure S1A), which may have five residues participating in PK2a. The 3′ residue of the H3 loop sequence involved in PK2a (C650), along with three downstream residues, can also putatively pair with sequences two nucleotides upstream of the Bridge to form a local pseudoknot (PK2b, light green, Figure 7A). The proposed overlapping usage of C650 by both PK2a and PK2b (Figure 7B, [X], in red) suggests that these pseudoknots do not exist simultaneously.

For simplicity, we labeled the H3 loop sequences, Domain 2 pairing partner sequence and Domain 3 pairing partner sequence, as [X], [Y] and [Z], respectively (Figure 7A). To examine the validity of PK2a and PK2b, mutations were introduced into [X] (m11A) or [Y] (m11B) to disrupt PK2b but not PK2a (Figure 7C, left). Frameshifting was reduced to 40 or 53% of WT when m11A or m11B were present, respectively (Figure 7D, left). Together, the compensatory mutations (m11C), which changed an A:U pair to a G:C pair, enhanced frameshifting to 82% of WT, supporting the existence of this local pseudoknot. Noteworthy for this set of alterations was that standard deviations for p81 levels were much larger than normal, suggesting that transcripts containing these mutations might not be adopting a uniform conformation.

Both single and double mutations were generated to evaluate PK2a (Figure 7C, middle). Single alternations in [X] and [Z] that disrupted PK2a but not PK2b (m12A and m12B, respectively) were highly detrimental, reducing frameshifting to background levels, whereas combining the two alternations, which would convert a G:C pair to a C:G pair, enhanced frameshifting to 82% of WT levels (m12C; Figure 7D, middle). Converting two C residues in [X] to Gs and two G residues in [Z] to Cs (m13A and m13B) also reduced frameshifting to background levels. The four alternations combined (m13C) restored frameshifting to WT levels. These results strongly support PK2a as a critical component of the active FSE, whereas PK2b may be functioning in a supporting role to assist in the formation of PK2a.

One unusual result from these alterations was finding that single and double mutations in the Domain 3 [Z] sequence (m12B and m13B) reduced translation of p21 by at least 75% when levels should have been significantly higher than WT due to reduced frameshifting. Compensatory mutations (m12C and m13C) increased translation of p21 to greater than WT levels (283 and 153%, respectively). This indicates that the specific [Z] alterations were not directly responsible for reduced translation. Rather, the reduction in translation may have resulted from a portion of the H3 loop sequence being newly accessible to a detrimental alternative pairing that disrupts the functioning of the 3′ CITE or other elements necessary for efficient translation. Why translation was enhanced by the compensatory mutations is not currently understood.

Residue C650 in H3 apical loops of all Class 2 ulaRNAs is shared by both PK2a and PK2b (Figure 7B, [X], in red). To explore the consequence of mutating this residue, C650 was changed to either a G (m14A) or an A (m15A). Both of these alterations, which should affect both PK2a and PK2b, reduced frameshifting by 66 and 73%, respectively, suggesting that critical PK2a was still forming in most of the transcripts. Altering the corresponding residue in [Y] to a C (m14C), or U (m15B), which should only disrupt PK2b, unexpectedly reduced frameshifting to background levels despite maintaining WT PK2a. This was in contrast to m11B, which altered a different residue in [Y] and maintained 40% frameshifting. Mutating the C650 pairing partner in [Z] to a C (m14B) or U (m15C), which should disrupt PK2a but not PK2b, reduced frameshifting to background levels. Curiously, reduced translation was not observed for these [Z] sequence alterations (m14B, m15C). Reestablishing full base-pairing for PK2a (C:G to G:C [m14E] or A:U [m15E]), but not for PK2b, enhanced frameshifting to 20 or 159% of WT, respectively, indicating that a weaker base-pair in PK2a is preferred when PK2b is destabilized. Reestablishing full base-pairing for PK2b only (G:C [m14D], A:U [m15D]) kept frameshifting at background levels, consistent with PK2a being the primary pseudoknot for -1 PRF. Reestablishing full base-pairing for both PK2a and PK2b (M14F and m15F), however, did not restore frameshifting. Interestingly, most of these C650 alterations (or its pairing partners) resulted in substantial increases in translation of p21, as much as 6-fold higher (m15D) than WT, accompanied by substantial variations in the data. Altogether, these results suggest that: (i) both PK2a and PK2b are involved in -1 PRF in CY1; (ii) PK2a is more critical for active frameshifting; (iii) the identity of the residue at position 650 is important and (iv) an unknown connection exists between translation from the 5′ end and the recoding site. In addition, we suggest that PK2a and PK2b cannot form simultaneously because of the shared C650 nucleotide, and that PK2b may support the formation of PK2a.

### Two tertiary interactions destabilize the H2 hairpin in the FSE and may be mechanistically connected

Ribosomes contain helicase activity (47,48) that unwind RNA structures so that the mRNA can thread through the ribosome during normal translation. One question that has not been adequately addressed is how a paused, frameshifting ribosome, following internal conformational changes that relocate the P-site and A-site tRNAs into the -1 frame, is able to unwind the FSE road block that prompted the initial ribosome pausing. In addition, although recent studies have clarified the importance of FSE conformational heterogeneity for frameshifting efficiency (27,49), whether a trigger exists that promotes these conformational changes remains undetermined.

It has been proposed that the ribosome itself melts out the first few base-pairs of the FSE so that alternative structures can be adopted (16,50). We decided to investigate a supplementary possibility: whether *cis-*acting, tertiary interactions destabilize the FSE following the frameshift allowing the ribosome to continue translating in the -1 frame. Using the Pairing Tool in RNAcanvas, two well-conserved, putative tertiary interactions were identified between upstream sequences and sequences on both sides of the critical central S2 stem in FSE H2 (Figure 5, Supplementary Figure S4, in blue and red). One of these tertiary interactions (PK3, in blue) connects five or six residues in the apical loop of a simple hairpin (H4), located just upstream of a conserved Y-shaped structure, with the 5′ side of H2, extending upward from the upper portion of S2 into the central interior bulge loop. Curiously, for teosinte and maize umbra-like viruses (TULV and MULV), the six-residue sequence in H2 that is complementary to the H4 apical loop is on the 3′ side of H2 (Supplementary Figure S4).

**Figure 8.**
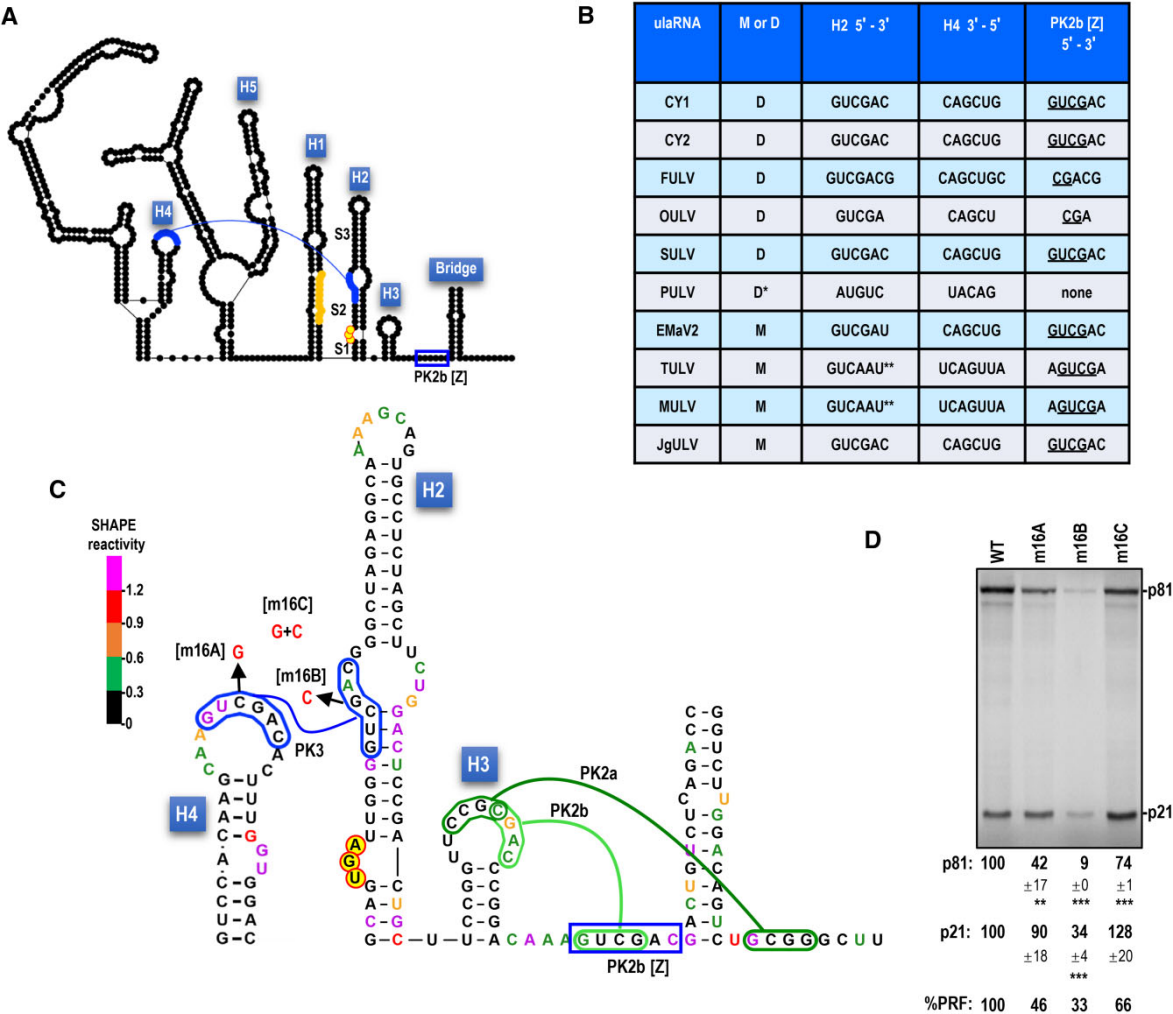
PK3 long-distance interaction is supported by compensatory mutations. A. Proposed PK3 tertiary interaction (in blue) between the apical loop of H4, located just upstream of a Y- shaped structure conserved in all ulaRNAs, and sequence on the 5′ side of H2 that includes the central symmetrical loop extending downward into S2. A second pairing partner for the H4 PK3 sequence is present in most Class 2 ulaRNAs overlaps the PK2b [Z] sequence just upstream of the Bridge (boxed in blue). Bases are colored by SHAPE structure probing. B. Table showing all Class 2 ulaRNA PK3 pairing partners in H2 and H4, as well as the possible additional pairing partner in the PK2b [Z] sequence (underlined sequence is involved in PK2b). No potential pairing partner for the H4 sequence is discernable for PULV in the PK2b [Z] location. Single asterisk denotes that PULV occupies a different clade from the other dicot-infecting Class 2 members. Double asterisks denotes that this sequence in H2 is in the S3 stem on the opposite site from the other PK3 interactions. C. Nucleotides are colored by SHAPE reactivity. Mutations (in red) that disrupt PK3 are shown. D. *In vitro* translation of full-length WT CY1 (WT) or mutant transcripts in WGE. Data are averages of at least three independent experiments and are normalized to levels of WT translation. Standard deviations are shown. One-way ANOVA was performed using GraphPrism software. *, ** and *** are *P* values of 0.01, <0.01 and <0.0001, respectively. PRF values are a percentage of the WT CY1 level.

PK3 in 5 Class 2 ulaRNAs (including CY1) is composed of identical H4 apical loop sequences (5′GUCGAC) (Figure 8A and B). Changing the middle C to a G (m16A) reduced frameshifting by 54%, suggesting a connection between H4 and -1 PRF. Altering the corresponding H2 residue from a G to a C (m16B), reduced translation by at least 66% and the frameshifting rate by 67%. Note that while this altered residue is within the internal loop of H2, it might still be affecting the critical S2 stem (see Figure 4, m6A, m6B, m6C). Combining H4 and H2 mutations, which were designed to be compensatory (m16C), restored translation to at least WT levels and improved the frameshifting rate to 66% of WT. The restoration of translation and enhanced frameshifting rate of m16C compared with m16B strongly suggest that m16A and m16B are compensatory. When combined with the strong conservation of PK3 in all ulaRNAs (see Supplementary Figures S5 and S6 for Class 3 and Class 1 ulaRNAs, respectively), these results support the existence of this pseudoknot. However, the critical necessity of the H2 S2 stem for frameshifting may be complicating interpretation of the effect of the single and compensatory PK3 mutations on frameshifting. Interestingly, a second possible H4 loop pairing sequence can be found for most of the Class 2 ulaRNAs in a location that overlaps with the PK2b [Z] sequence (Figure 8A and B, blue box).

**Figure 9.**
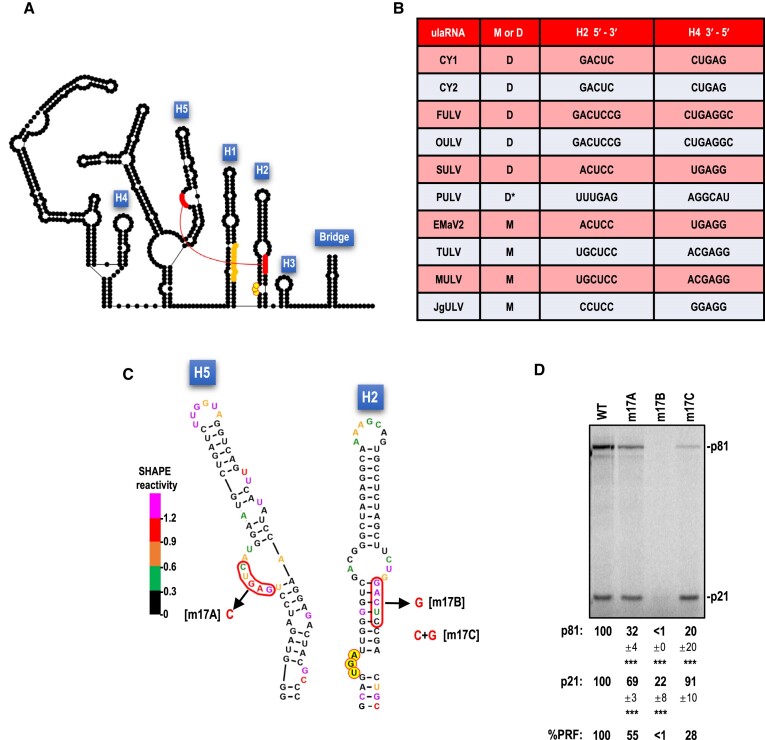
PK4 long-distance interaction is supported by compensatory mutations. A. Proposed PK4 tertiary interaction (in red) between a bulge loop in H5, located just downstream of the Y-shaped structure, and sequence on the 3′ side of the H2 S2 stem. Unlike PK3, no additional pairing partner for the H5 sequence is discernable. B. Table showing all Class 2 ulaRNA PK4 pairing partners in H2 and H5. C. Nucleotides are colored by SHAPE reactivity. Mutations (in red) that disrupt PK4 are shown. D. *In vitro* translation of full-length WT CY1 (WT) or mutant transcripts in WGE. Data are averages of at least three independent experiments and are normalized to levels of WT translation. Standard deviations are shown. One-way ANOVA was performed using GraphPrism software. *, ** and *** are *P* values of 0.01, <0.01 and <0.0001, respectively. PRF values are a percentage of the WT CY1 level.

Searching for pairing partners on the 3′ side of H2 S2 led to the identification of PK4, proposed to connect this sequence with an asymmetric bulge loop in hairpin H5 (Figure 5, in red; Figure 9A; Supplementary Figure S4, in red). PK4 is postulated to occur only in Class 2 ulaRNAs, which are the only ulaRNAs predicted to contain H5 associated with their conserved Y-shaped structures. Putative PK4 consists of five to seven Watson–Crick base pairs with the exception of PULV, which contains G:U base-pairs (Figure 9B). The H5 bulge loop sequence in CY1 (5′ GAGUC) and the putative pairing partner in the H2 stem are strongly reactive with the SHAPE reagent (Figure 9A), suggesting that if this pseudoknot exists, it is not in the *in vitro* synthesized transcripts subjected to SHAPE. Altering the central G in the PK4 H5 sequence to a C (m17A) reduced frameshifting by 45% (Figure 9C), similar to the effect of altering the H4 apical loop and PK3. Changing the corresponding residue in the H2 S2 stem from C to G (m17B) reduced translation of p21 by 78%, which was again similar to the effect on translation of the H2 alteration that would disrupt PK3 (m16B). This result for m17B was unexpected, as the two-residue alteration in m6B (Figure 4) included this C to G alteration and there was no detrimental effect on translation. Combining both m17A and m17B (m17C), which should restore PK4 at the expense of destabilizing S2, restored p21 translation, with frameshifting now at 28% of WT. Note that for both PK3 and PK4 alterations, the compensatory mutations were engineered into the m16B and m17B constructs, indicating that loss of translation for m16B and m17B was not due to spurious mutations. Restoration of translation by the compensatory PK4 alterations, similar to the compensatory mutations for PK3, combined with strong conservation of the interaction in Class 2 ulaRNAs, supports the presence of PK4. In addition, the similar results for the single and compensatory mutations that disrupt and reform PK3 and PK4 suggest that these pseudoknots may be mechanistically connected. Altogether, these results support the presence of PK3 and PK4, which when present would significantly disrupt the structure of the critical H2 S2 stem and destabilize the FSE.

### An alternative, inactive structure conserved in Class 2 ulaRNAs supports the SHAPE structure data

**Figure 10.**
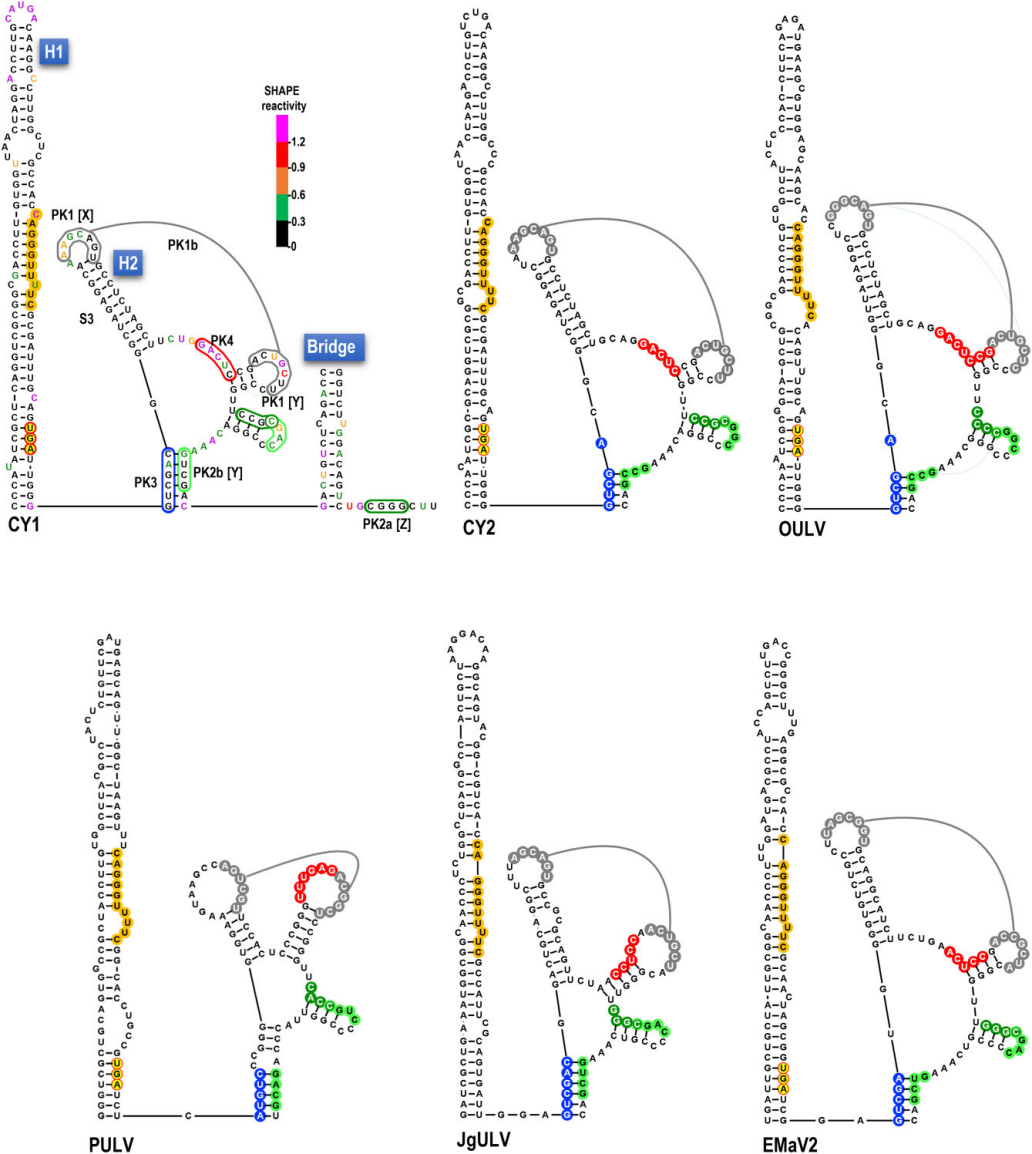
Alternative structures for Class 2 ulaRNA FSEs. Conserved alternative structure is shown for CY1 and selected Class 2 ulaRNAs. These structures contain the alternative, extended form of H1 shown in Figure 3, right, and Supplementary Figure S3. Sequences that participate in tertiary interactions in the active structure are color coded: PK1b, gray; PK2a, dark green; PK2b, light green; PK3, blue; PK4, red. Nucleotides in CY1 are colored according to SHAPE reactivity, which offer better support for this structure than the structure shown in Figure 2. PK1b is compatible with this structure in all Class 2 ulaRNAs.

If the conserved, alternative structure for H1 forms (Figure 3A, right), the remaining FSE nucleotides can adopt an alternative structure for CY1 that strongly supports the CY1 SHAPE data (Figure 10, top left) and is conserved in all other Class 2 ulaRNAs. We propose that only the PK1b pseudoknot is present in this alternative structure, in the form of a kissing-loop interaction between the H2 apical loop and the apical loop of a new hairpin. H3 and downstream residues up to the Bridge are proposed to rearrange into a stem composed of PK3 sequences (in blue) paired with PK2b [Y] sequences. As this conserved alternative structure eliminates essential -1 PRF elements H2 S2 as well as PK1a and PK2a, we propose that it represents an inactive form of the FSE that is present in most *in vitro* synthesized transcripts. Interestingly, the Domain 3 PK2a pairing partner (PK2a [Z] sequence), and most of the H4 apical loop PK3 pairing sequence, are unreactive with the SHAPE reagent, suggesting that they may also have alternative unidentified pairing partners. PK4 sequences in H2 and H5 are strongly reactive with the SHAPE reagent, and thus are proposed to be unstructured in the alternative FSE in CY1. Interestingly, the H2 PK4 sequences for several Class 2 ulaRNAs (in red) are not available for pairing, supporting the possibility that PK4 does not form in this structure.

### Model for frameshifting in Class 2 ulaRNAs

**Figure 11.**
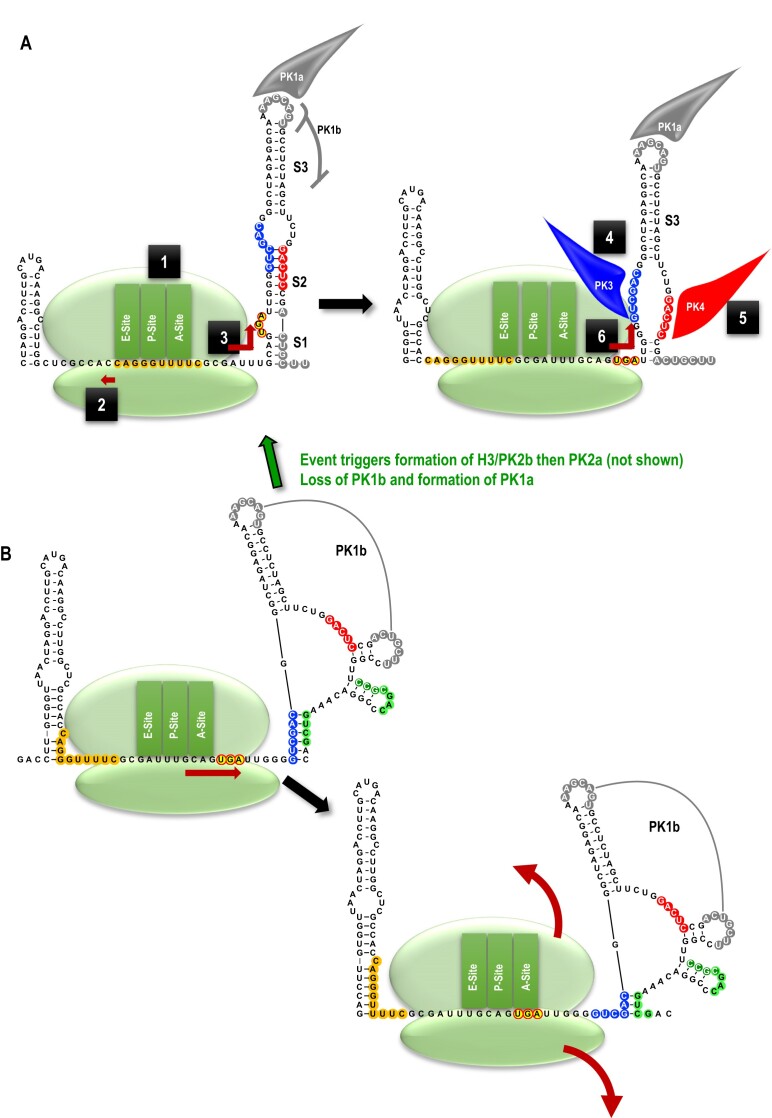
Model for -1 PRF in Class 2 ulaRNAs. A. Frameshifting using the active FSE structure. B. Inactive structure promoting translation termination and conversion between inactive and active structures. See text for explanation.

A model for CY1 frameshifting is shown in Figure 11. We propose the existence of two major structures for the FSE of CY1 and other Class 2 ulaRNAs: (i) an active structure (shown in its entirety in Figure 5) with a shorter H1, H2 with all three stems and apical loop sequences engaged in PK1a, H3 engaged in PK2b and then PK2a, with the PK2b [Y] sequence possibly blocked by pairing with the H4 apical loop; and (ii) an inactive structure, with an extended H1, H2 containing only the upper S3 stem and the H2 apical loop engaged in PK1b (as shown in Figure 10). As SHAPE data support the latter structure, this suggests that the majority of the *in vitro* synthesized transcripts contain the inactive structure. This is similar to the ribosomal readthrough element in carmovirus TCV, whose inactive structure dominated in transcripts and *in vivo* (6). In addition, we propose intermediate conformations of the FSE that form when the inactive structure transitions to the active structure, and during the process of frameshifting, when the ribosome translates in the -1 frame through the FSE.

Figure 11A depicts the model for how ribosomes frameshift in the presence of the active FSE structure. The following numbers refer to the numbers in the figure: (1) the stable FSE active structure acts as a road block, stalling the ribosome with the A- and P-site tRNAs occupying the slippery site. (2) The ribosome shifts backward by 1 nt, repositioning the tRNAs in the -1 frame. (3) Using its endogenous helicase activity, the ribosome proceeds forward, unwinding the H2 S1 bottom stem. (4) In an unknown manner, the unwinding of S1 makes the H2 PK3-pairing sequence available, leading to the formation of PK3. PK3 causes the top portion of the H2 S2 stem to unzip, opening up the PK4 partner sequence. (5) Formation of PK4 then unzips most of the remaining S2 stem. (6) With the FSE destabilized, the ribosome can now translate through the FSE without impediment, leading to synthesis of the viral RdRp.

As shown in Figure 11B, the FSE inactive structure does not block the ribosome, which translates through the slippery site and terminates at the p21 UGA. Furthermore, we propose that transitioning between inactive and active structures (green arrow) is caused by formation of H3 and PK2b, which then supports the formation of PK2a, replacing PK2b. This intermediate FSE conformation allows the PK1b [Y] sequence to form the S1 stem, leading to the formation of the S2 stem. Without PK1b, the H2 apical loop connects with 3′ terminal sequences in PK1a, which stabilizes the active structure by keeping PK1b from forming.

Whereas the current *in vitro* derived data are consistent with these models, many questions remain. For example, why do single mutations in H2 that disrupt PK3 (m16B) and PK4 (m17B) significantly reduce translation of p21 whereas many mutations in C650 and its partner sequences significantly enhance p21 translation? Previous analysis of the CY1 3′ CITE revealed that a number of alterations in the CITE lower stem significantly affected frameshifting (37), suggesting a possible connection between the FSE and the 3′ CITE. Additionally, what keeps PK3 from forming prematurely (i.e. before the frameshift has taken place)? Furthermore, is there an event that triggers the conformational switch between inactive and active states? Or, are these conformations co-existing in equilibrium and constantly switching, with the inactive structure present ∼71% of the time, while the active structure is present around 29% of the time? Additional work is needed to answer these and other questions that will enhance our understanding of Class 2 ulaRNA frameshifting.

### Class 3 and Class 1 ulaRNAs frameshifting structures

We also examined sequences in the vicinity of the recoding sites for Class 3 and Class 1 ulaRNAs, which are more closely related to each other than they are to Class 2 ulaRNAs (Supplementary Figure S1B). As shown in Supplemental Figures S5 and S6, all Class 1 and 3 ulaRNAs are capable of forming PK1b, highlighting the critical importance of this pseudoknot, however, only Class 3 GULV has a putative PK1a [Z] sequence near the 3′ end of its genome. This suggests that the other Class 1 and Class 3 ulaRNAs employ a different mechanism for preventing PK1b in their active conformation. Both Class 1 and 3 ulaRNAs have sequences capable of forming PK3 that occupy the same locations in H4 and H2 as the Class 2 ulaRNAs, signifying an important role for this highly conserved pseudoknot. By contrast, PK4 is not discernable for any Class 1 or 3 ulaRNA, suggesting an alternative mechanism might be present to help destabilize the FSE. Interestingly, all Class 1 and 3 ulaRNAs have a new pseudoknot (PK2c) comprising 8–10 base-pairs between their H3 apical loops and the bottom portion of H1. In addition, whereas Class 3 ulaRNAs have a putative Domain 2 Bridge and PK2a [Z] sequence just past the bridge (Supplementary Figure S5), this interaction cannot be found for Class 1 ulaRNAs. Furthermore, Class 3 PK2a are composed of 5–6 Watson–Crick base-pairs compared with four base-pairs for Class 2 members other than PULV. In addition, PK2b is either not present or poorly supported in non-identical locations for Class 3 ulaRNAs (Supplementary Figure S5A), and is not found for any of the Class 1 ulaRNAs. These findings suggest that different classes of ulaRNAs have evolved diverse mechanisms to transition between different FSE conformations.

## CONCLUSIONS

In conclusion, we have discovered multiple tertiary interactions that affect the structure of the highly efficient CY1 FSE covering nearly three-quarters of its genome. We propose that the two sets of incompatible interactions (PK1a/PK1b; PK2a/PK2b) are involved in transitioning between inactive and active structures. More importantly, the discovery of PK3 and PK4 suggests that *cis-*acting interactions are needed to destabilize the CY1 FSE before a frameshifted ribosome can proceed with translation. None of the long-distance interactions (PK1a, PK2a, PK3 and PK4) would have been discernable if the local FSE structure had been studied in a reporter construct or via optical tweezers, which would have significantly affected any drawn conclusions. Our results strongly support the gathering consensus (4,8,22,30) that FSEs must be studied within their native context to more accurately understand the mechanism of frameshifting.

## Supplementary Material

gkad744_Supplemental_File

## Data Availability

Data calculations for all WGE experiments are available at https://doi.org/10.6084/m9.figshare.23648727.
