# Supplementary material for: -1 Programmed ribosomal frameshifting in Class 2 umbravirus-like RNAs uses multiple long-distance interactions to shift between active and inactive structures and destabilize the frameshift stimulating element
Source: Nucleic Acids Res. 2023 Sep 23;51(19):10700–18. doi: 10.1093/nar/gkad744 (PMC10602861; doi:10.1093/nar/gkad744)
Supplement: gkad744_Supplemental_File [file gkad744_supplemental_file.pdf]

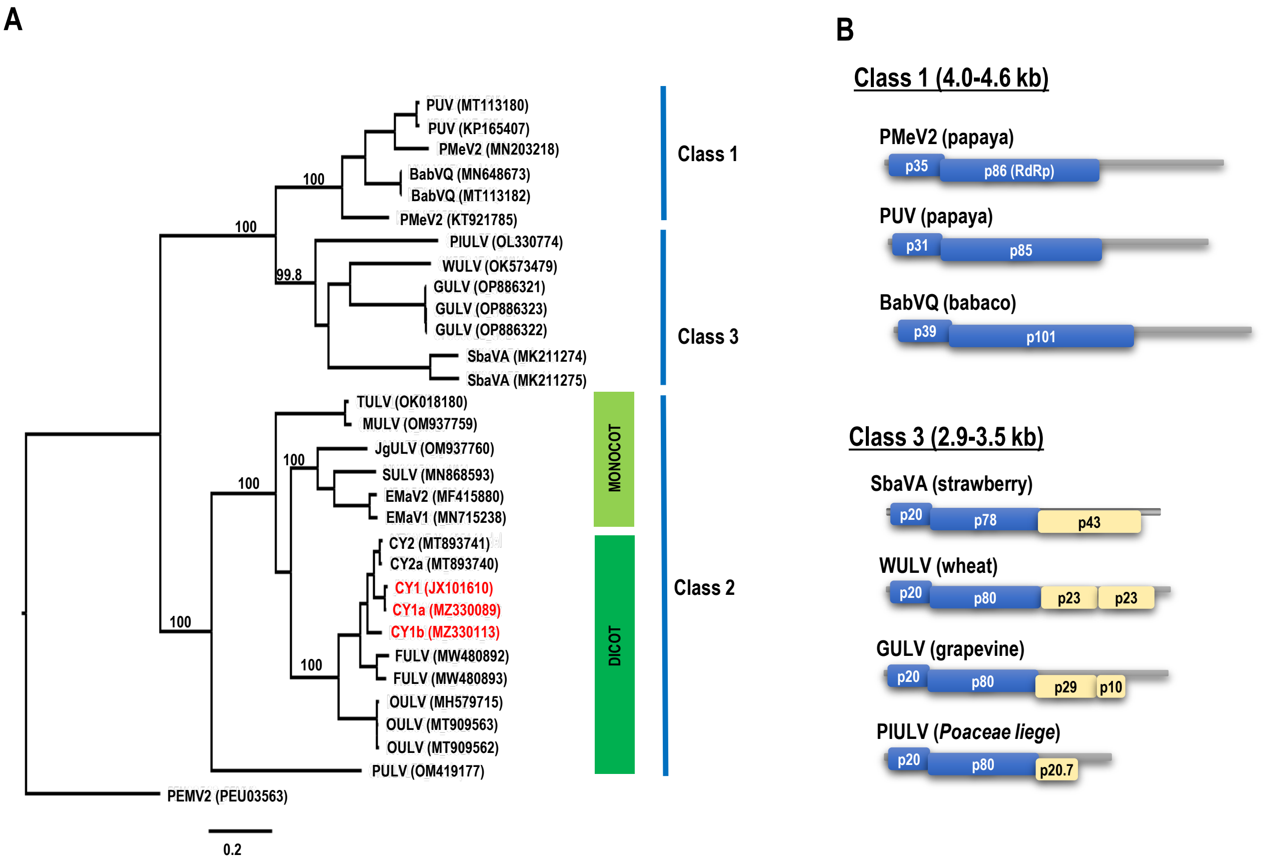

C

Name	Class	Slippery sequence Stop codon
		X XXY YYZ
CY1	2 (D)	CAG GGU UUU CGC GAU UUG CAG UGA
CY2	2 (D)	CAG GGU UUU CGC GAU UUG CAG UGA
FULV	2 (D)	CAG GGU UUU CAC AGU UUG CAG UGA
OULV	2 (D)	CAG GGU UUU CAC AGU UUG CAG UGA
SULV	2 (D)	CAG GGU UUU CGG AGU UCG CGG UGA
PULV	2 (D*)	CAG GGU UUU CGG CAC CUG CCG UGA
JgULV	2 (M)	CAG GGU UUU CGC CAU UCG CAG UGA
EMaV2	2 (M)	CAG GGU UUU CGC AAC UAG CGG UGA
TULV	2 (M)	CAG GGU UUU CGG CAU UAG CGG UGA
MULV	2 (M)	CAG GGU UUU CGG CAC UAG CGG UGA
GULV	3	CCG GUU UUU CGC GAU GGG UGG UGA
SbaVA	3	CCG GGA AAC GAC GAU GGG UGG UGA
PIULV	3	UCG CGU UUU CAC AGU GGG UGG UAA
WULV	3	CUG GGU UUU CCG AGU GGG UGG UAA
BabVQ	1	CCG UCU UUU CUG GUU GGC CGG UAA
PMeV2	1	AAG CCU UUU UAG GAU GGC AGG UGG
PUV	1	CAU CCU UUU CUG GUU GGC CGG UAA
PEMV2	Umbra virus	GCG GAU UUU UGG UAG

Supplemental Figure 1. Slippery sequences and first ORF termination codons for all ulaRNAs. A. Maximum likelihood phylogenetic tree based on RdRp amino acid sequences. Branch numbers indicate bootstrap support in percentage out of 1000 replicates. The scale bar denotes protein substitutions per site. Umbravirus pea enation mosaic virus 2 (PEMV2) was used as an outgroup and rooted to it. SbaVA, Strawberry virus A; GULV, grapevine umbra-like virus (1); WULV, Wheat umbra-like virus (2); PIULV, Poaceae liege umbra-like virus (3); PMeV2, Papaya meleira virus 2 (4); BabVQ, Babaco virus Q (5); PUV, Papaya umbravirus (6). See legend to Fig 1 for additional abbreviations. B. Gene organization of Class 1 and Class 3 ulaRNAs. Replication proteins are in blue. Extra ORFs in Class 3 ulaRNAs are not apparently related to each other and likely represent different recombination events. C. Slippery sequences and downstream termination codons for the first ORF. Stop codons are underlined. "XXX" of the slippery sequences are in red. Class 2 monocot-infecting (M) or dicot-infecting (D) is indicated.

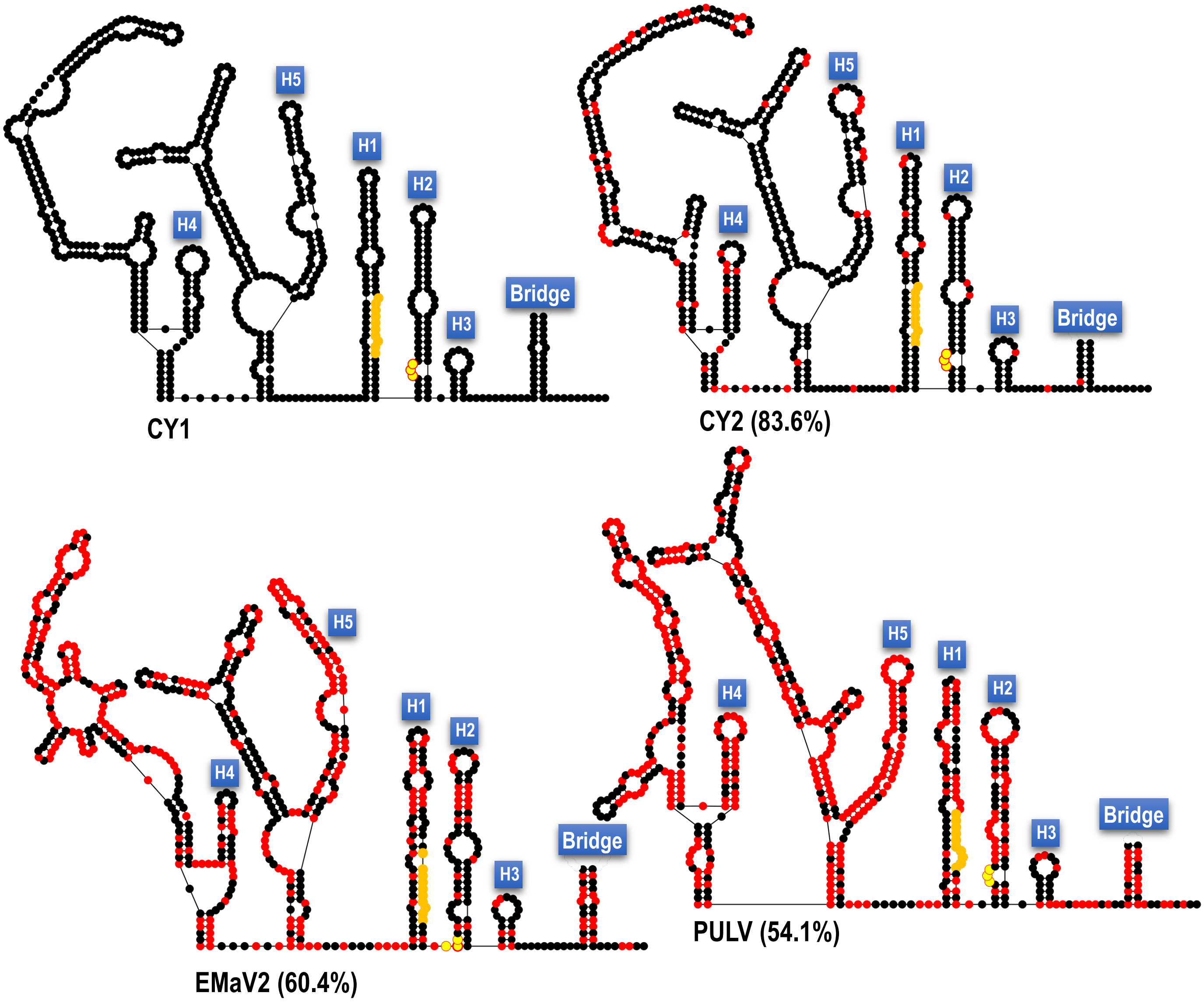

Supplemental Figure 2. Sequence similarity between CY1 and three Class 2 ulaRNAs. CY2 is the closest related ulaRNA. EMaV2 is a monocot-infecting ulaRNA. PULV is dicot-infecting but occupies its own clade (see SFig. 1A). Although not shown here, all other Class 2 ulaRNAs can form similar structures. Percentage sequence identity with CY1 is given. Each dot represents a nucleotide. Red dots denote sequence differences. Orange dots are the slippery sequence with three additional conserved residues. Yellow dots with red border are the termination codon. Bridge and hairpins are labeled as in Fig. 1C. Putative structures for CY2, EMaV2 and PULV are based on the known CY1 structure.

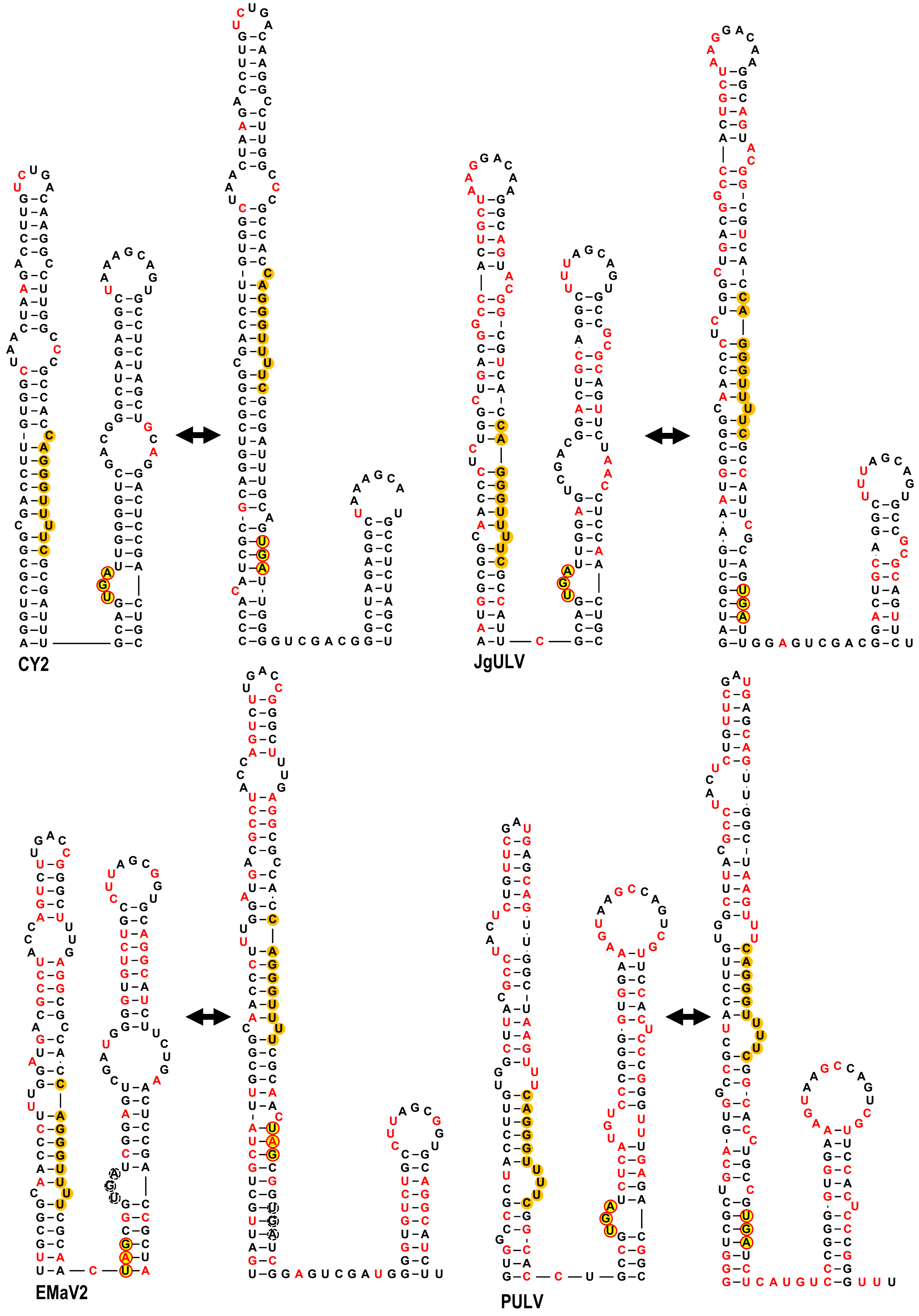

Supplemental Figure S3. Alternative conformation for H1 and H2 in selected Class 2 ularRNAs. CY2 is the closest relative to CY1; JgULV and EMaV2 infect monocots and PULV is the least similar Class 2 ularRNA. Nucleotides that differ with CY1 are in red. Slippery site with three additional fully conserved nucleotides is in orange. Stop codon for the first ORF is in yellow with a red border. Broken circled bases for EMaV2 are the unused UGA codon. The Class 2 ularRNAs not shown have very similar conformations of H1/H2.

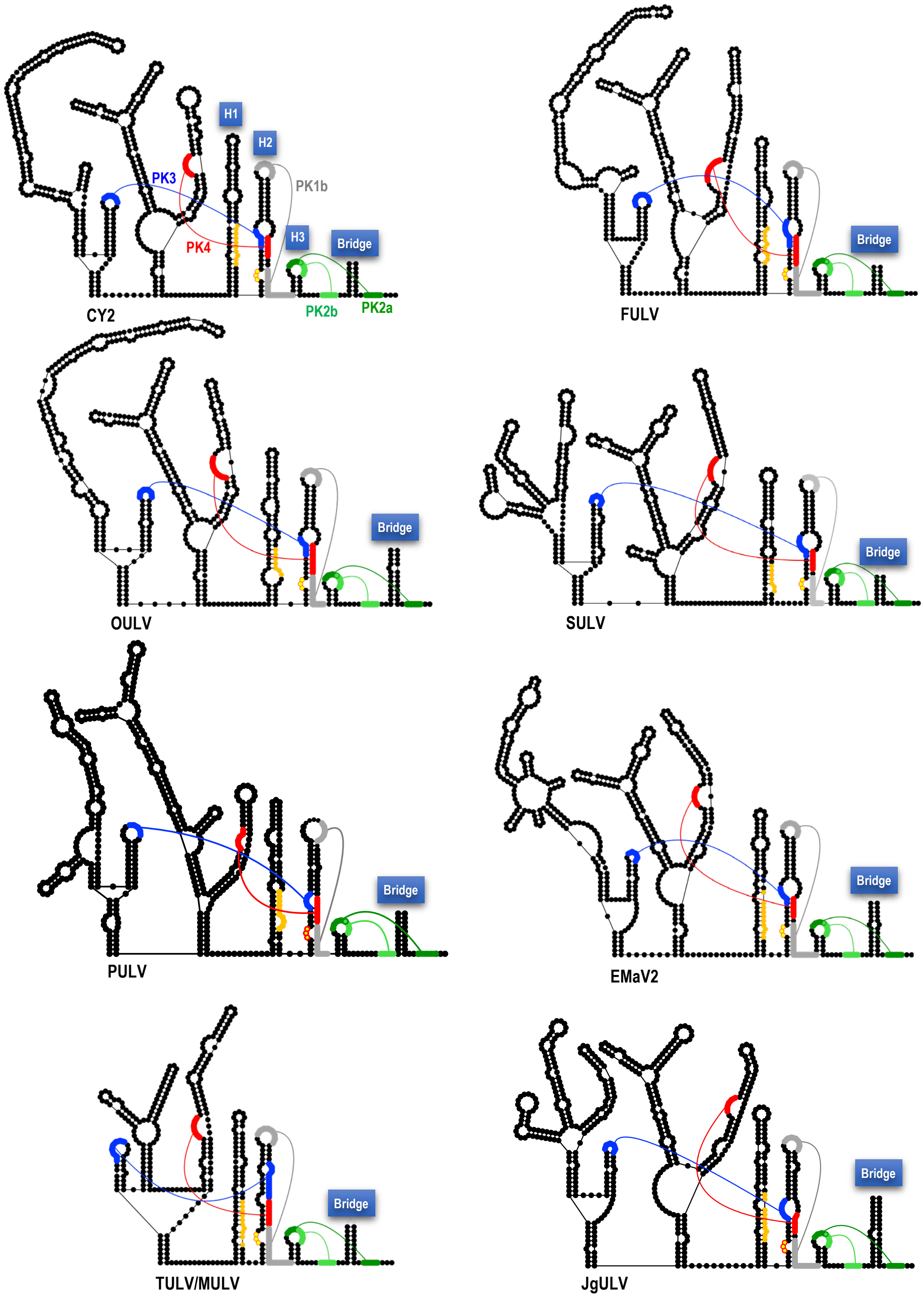

Supplemental Figure 4. Structures and proposed tertiary interactions in Class 2 ulaRNAs. Structures are drawn based on the known CY1 structure. Identity of the nucleotides involved in these tertiary interactions is shown for PK1b (and PK1a, not shown) in Figure 6B; PK2a and PK2b in Figure 7B; PK3 in Figure 8B; and PK4 in Figure 9B.

A

ulaRNA	PK1 [X] 5′ - 3′	PK1b [Y] 3′ - 5′	PK1a [Z] 3′ - 5′	
GULV	AGUUCUGCAGUC	GACGUCGG	UUAAGAC	
SULV	UCUUCGCCC	AGAAGUGGG	?	
PLULV	CCUGGAGCC	GGCCUCGG	?	
WULV	CGAAUGCCC	GCUUGCGG	?	
	PK2 [X] 5′ - 3′	PK2a [Y] 3′ - 5′	PK2b [Z] 3′ - 5′	PK2c [ZZ] H1 3′ - 5′
GULV	GCGCGCCACCC	CGCGCG	*GCGUG*	GUGGUGGG
SULV	GGGUGCACCACCC	CCCACG	*CAUG?*	GUGGUGGG
PIULV	UGCCACCC	CGGUGG	*?*	AUGGUGGG
WULV	GCUGCCACCC	CGACG	*GACGG*	AUGGUGGG
	PK3 H2 5′ - 3′	PK3 H4 3′ - 5′		
GULV	GCUCC	CGAGG		
SULV	AUUCCAG	UAAGGUC		
PIULV	UUUCC	AAAGG		
WULV	AGCUCC	UCGAGG		

B

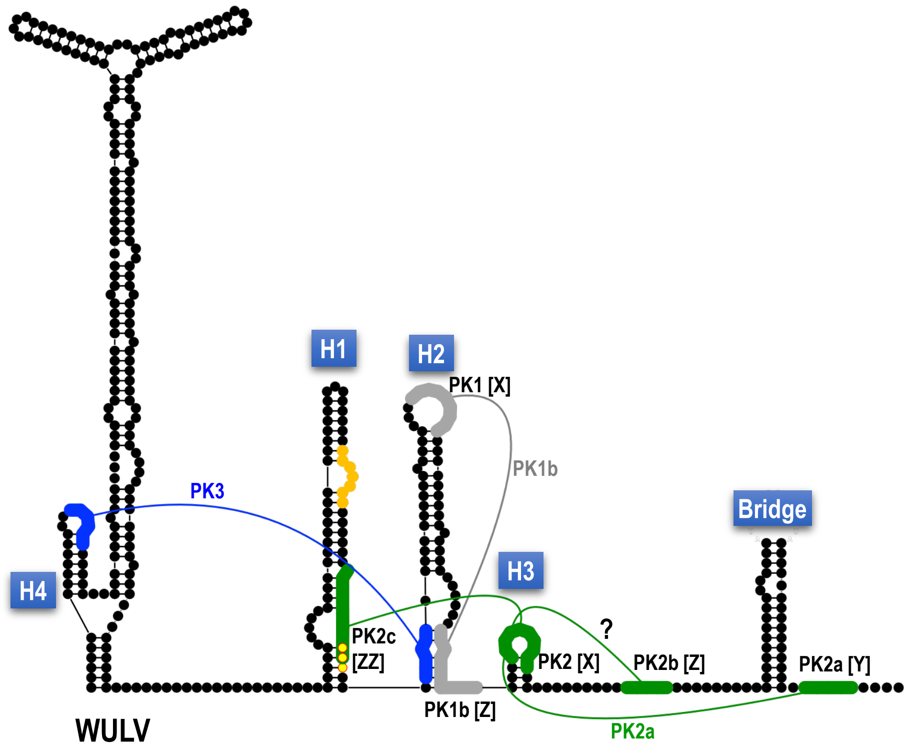

Supplemental Figure 5. Class 3 ulaRNAs contain sequences that can form some similar and some different tertiary interactions. A. Sequences of proposed tertiary interactions in Class 3 ulaRNAs. B. Putative structures of Class 3 ulaRNAs (continued on the following page). For WULV and SULV, PK3 is similar to Class 2, pairing the apical loop of the hairpin upstream of the conserved Y-shaped structure (H4) with sequences on the 5' side of the lower stem of H2. For the other two Class 3 ulaRNAs, the H4 pairing partner is in a similar location but not discernably part of the H2 stem. PK1b is similar to that of Class 2 ulaRNAs. However, which one exception, PK1a connecting sequences near the 3' end is not discernable. H3 apical loop has two pairing partners for all Class 3 ulaRNAs: a new pairing sequence within H1 that includes 2 or 3 of the stop codon nucleotides (PK2c); and PK2a, with complementary sequence on the other side of a similar long-distance bridging sequence. Interestingly, PK2a is more stable (5 to 6 bp) than for Class 2 ulaRNAs (4 bp). If the PK2b local pseudoknot exists, it is not in conserved locations and is not discernable for all Class 3 ulaRNAs. H5 is not present in Class 3 ulaRNAs, and there is no comparable PK4 interaction.

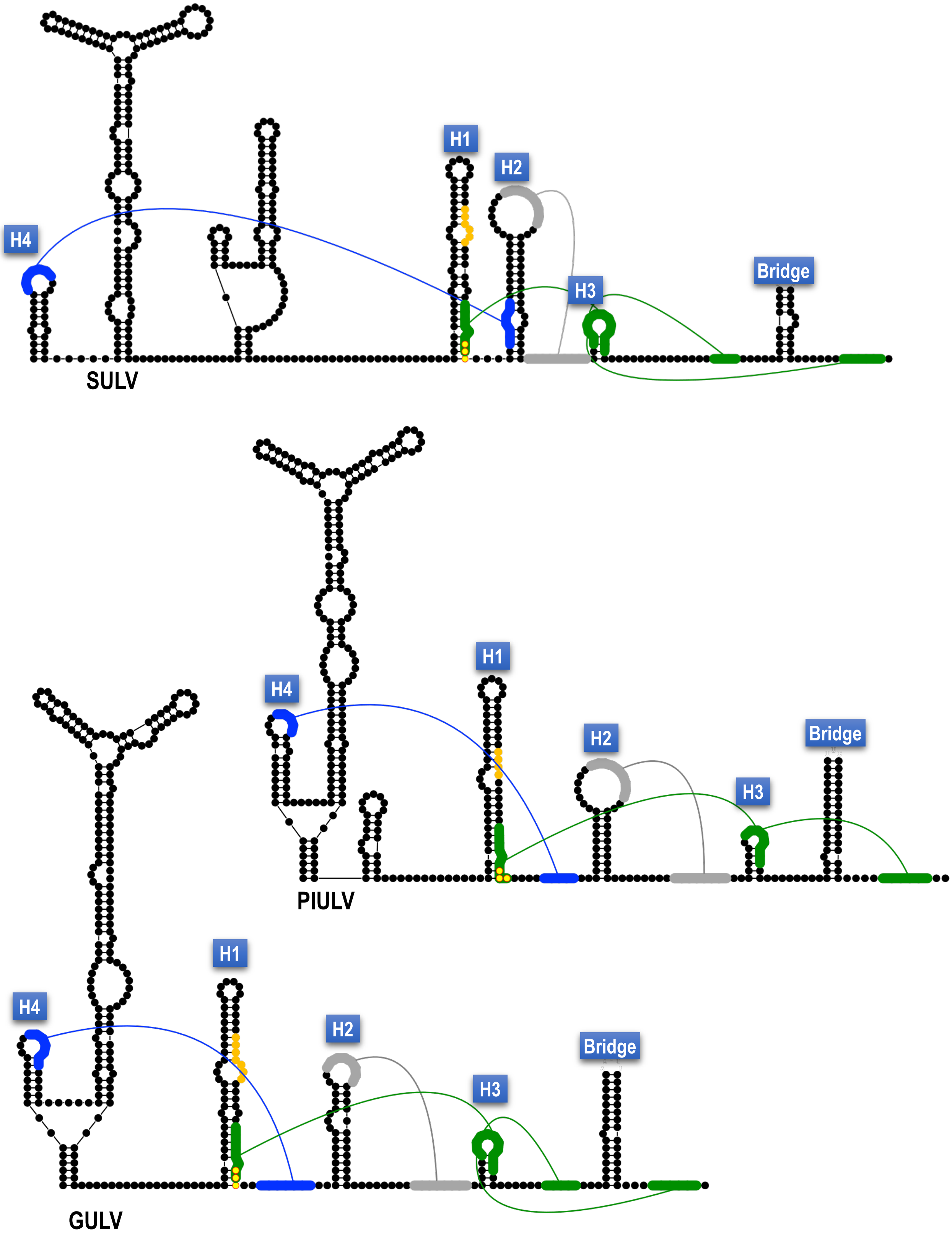

Supplemental Figure 5 (con't)

A

ulaRNA	PK1 [X] 5′ - 3′	PK1b [Z] 3′ - 5′
BabVQ	CGUAGUUCCCAA	GCAUCaAAGGGUU
PMeV2	GUUCGUAAU	CAAGCAUUA
PUV	GCAAUUCC	CGUUaAAGG
	PK2 [X] 5′ - 3′	PK2c [ZZ] 3′ - 5′
BabVQ	UACCGGCC	AUGGCCGG
PMeV	CUUGCGGGCC	GAAUGCCCGG
PUV	GAAUGGCCGG	CUUACCGGCC
	PK3 [H2] 5′ - 3′	PK3 [H4] 3′ - 5′
BabVQ	ACAGUUCCC	UGUCGAGGG
PMeV	CGUUUCCA	GCGAGGGU
PUV	ACGUUUCC	UGCGAGGG

B

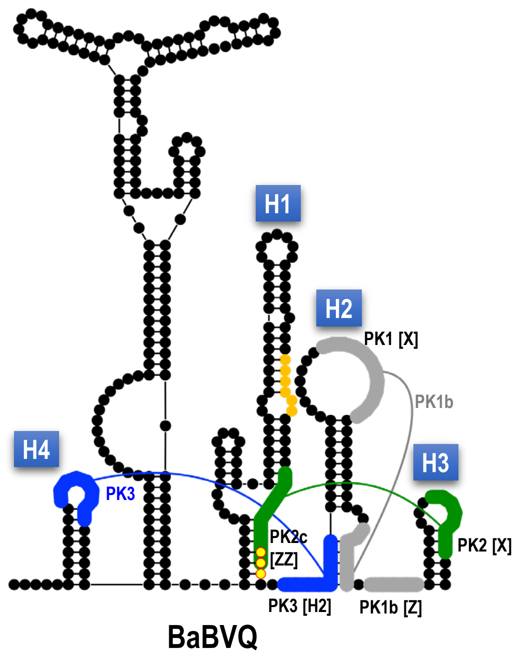

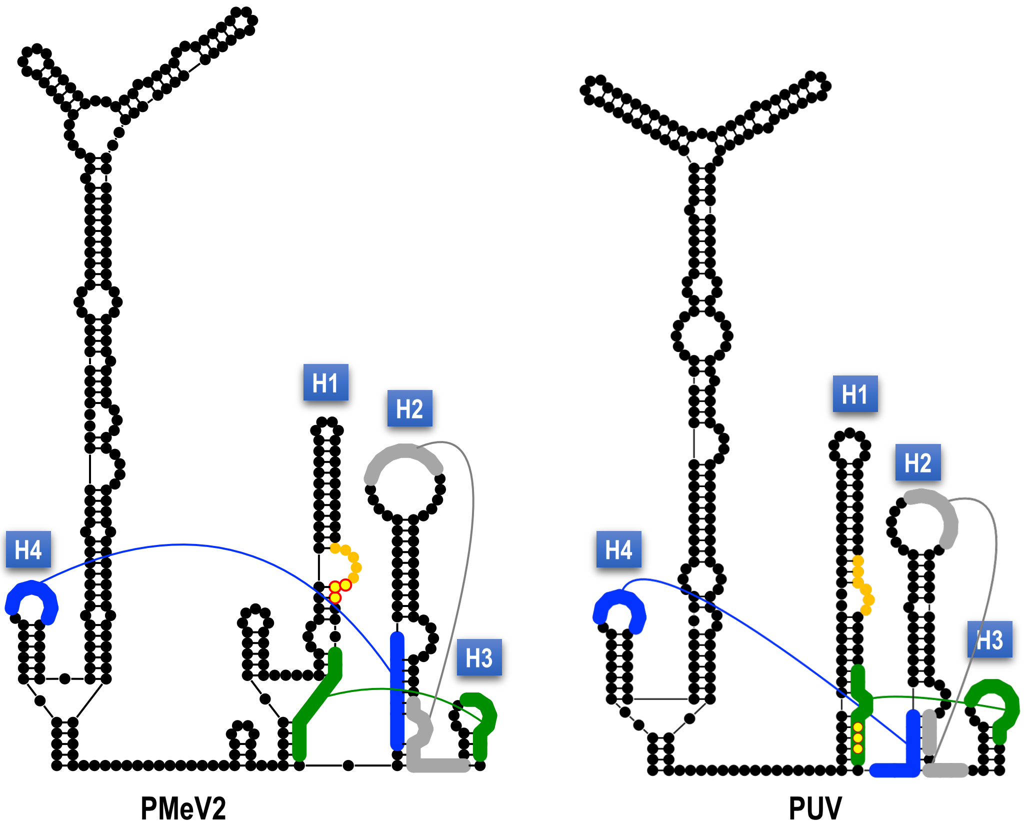

Supplemental Figure 6. Class 1 ulaRNAs contain sequences that can form some similar and some different tertiary interactions from Class 2 and 3. A. Sequences of proposed tertiary interactions in Class 1 ulaRNAs. B. Putative structures of Class 1 ulaRNAs. As with Class 2 and several Class 3 ulaRNAs, PK3 pairs the apical loop of the hairpin upstream of the conserved Y-shaped structure (H4) with sequences on the 5' side of the lower stem of H2. PK1b is similar to that of Class 2 and Class 3 ulaRNAs. However, as with most Class 3 ulaRNAs, no PK1a connecting with sequences near the 3' end was discernable. The new Class 3 H3 apical loop interaction with H1 (PK2c) was also present in all three Class 1 ulaRNAs, however no PK2a or PK2b was discernable.

References for Supplemental Figures

1. Shvets, D., Sandomirsky, K., Porotikova, E. and Vinogradova, S. (2022) Metagenomic analysis of ampelographic collections of dagestan revealed the presence of two novel grapevine viruses. *Viruses*, **14**.

2. Redila, C.D., Prakash, V. and Nouri, S. (2021) Metagenomics analysis of the wheat virome identifies novel plant and fungal-associated viral sequences. *Viruses*, **13**.

3. Maclot, F., Debue, V., Malmstrom, C.M., Filloux, D., Roumagnac, P., Eck, M., Tamisier, L., Blouin, A.G., Candresse, T. and Massart, S. (2023) Long-term anthropogenic management and associated loss of plant diversity deeply impact virome richness and composition of poaceae communities. *Microbiol Spect*, **11**.

4. Sa Antunes, T.F., Vionette Amaral, R.J., Ventura, J.A., Godinho, M.T., Amaral, J.G., Souza, F.O., Zerbini, P.A., Zerbini, F.M. and Bueno Fernandes, P.M. (2016) The dsRNA virus papaya meleira virus and an ssRNA virus are associated with papaya sticky disease. *PLoS One*, **11**.

5. Cornejo-Franco, J.F., Flores, F., Mollov, D. and Quito-Avila, D.F. (2021) An umbra-related virus found in babaco (Vasconcellea x heilbornii). *Arch Virol*, **166**, 2321-2324.

6. Quito-Avila, D.F., Alvarez, R.A., Ibarra, M.A. and Martin, R.R. (2015) Detection and partial genome sequence of a new umbra-like virus of papaya discovered in Ecuador. *Eur J Plant Path*, **143**, 199-204.
